# Gene Splicing of an Invertebrate Beta Subunit (LCavβ) in the N-Terminal and HOOK Domains and Its Regulation of LCav1 and LCav2 Calcium Channels

**DOI:** 10.1371/journal.pone.0092941

**Published:** 2014-04-01

**Authors:** Taylor F. Dawson, Adrienne N. Boone, Adriano Senatore, Joshua Piticaru, Shano Thiyagalingam, Daniel Jackson, Angus Davison, J. David Spafford

**Affiliations:** 1 Department of Biology, University of Waterloo, Waterloo, Ontario, Canada; 2 Institute of Genetics, School of Biology, University of Nottingham, Nottingham, United Kingdom; Indiana University School of Medicine, United States of America

## Abstract

The accessory beta subunit (Ca_v_β) of calcium channels first appear in the same genome as Ca_v_1 L-type calcium channels in single-celled coanoflagellates. The complexity of this relationship expanded in vertebrates to include four different possible Ca_v_β subunits (β_1_, β_2_, β_3_, β_4_) which associate with four Ca_v_1 channel isoforms (Ca_v_1.1 to Ca_v_1.4) and three Ca_v_2 channel isoforms (Ca_v_2.1 to Ca_v_2.3). Here we assess the fundamentally-shared features of the Ca_v_β subunit in an invertebrate model (pond snail *Lymnaea stagnalis*) that bears only three homologous genes: (LCa_v_1, LCa_v_2, and LCa_v_β). Invertebrate Ca_v_β subunits (in flatworms, snails, squid and honeybees) slow the inactivation kinetics of Ca_v_2 channels, and they do so with variable N-termini and lacking the canonical palmitoylation residues of the vertebrate β2a subunit. Alternative splicing of exon 7 of the HOOK domain is a primary determinant of a slow inactivation kinetics imparted by the invertebrate LCa_v_β subunit. LCa_v_β will also slow the inactivation kinetics of LCa_v_3 T-type channels, but this is likely not physiologically relevant *in vivo*. Variable N-termini have little influence on the voltage-dependent inactivation kinetics of differing invertebrate Ca_v_β subunits, but the expression pattern of N-terminal splice isoforms appears to be highly tissue specific. Molluscan LCa_v_β subunits have an N-terminal “A” isoform (coded by exons: 1a and 1b) that structurally resembles the muscle specific variant of vertebrate β1a subunit, and has a broad mRNA expression profile in brain, heart, muscle and glands. A more variable “B” N-terminus (exon 2) in the exon position of mammalian β3 and has a more brain-centric mRNA expression pattern. Lastly, we suggest that the facilitation of closed-state inactivation (e.g. observed in Ca_v_2.2 and Ca_v_β_3_ subunit combinations) is a specialization in vertebrates, because neither snail subunit (LCa_v_2 nor LCa_v_β) appears to be compatible with this observed property.

## Introduction

Unique ancillary beta subunits are identifiable in the proteosomal complex with different voltage-gated ion channels including K [1]–[3], Na^+^ [4]–[6] and Ca^2+^ [7]–[9] channels. These accessory subunits are known to promote the membrane expression and trafficking of ion channel complexes, as well as to modify the biophysical features of voltage-gated ion channels [7]–[9]. Sodium channels (Na_v_1.x) and calcium channels (Ca_v_1.x, Ca_v_2.x, Ca_v_3.x) bear a common structural template of four repeat domains of six transmembrane helices each, but they have distinct ancillary subunits which are known to regulate them [7]–[9]. Sodium channel beta subunits (Na_v_β) evolved separately in different animal groups such as insects, snails and vertebrates. TipE/Teh are insect Na_v_β subunits and have a likeness to (Slo/BK) Kvβ subunits with EGF-like domains [4], while gastropod snails Na_v_β subunits is a CUB domain containing protein family [10], while vertebrate Na_v_β subunits are CaM-like with a V-set Ig extracellular loop [5]. Calcium channel beta subunits (Ca_v_β) have common homologs in animal groups rooted in genomes of single cell organisms like coanoflagellates, which also possess an L-type calcium channel (Ca_v_1) homolog [11]. From a likely primordial template of one Ca_v_1 and Ca_v_β subunit in coanoflagellates [12], emerged the complexity in numbers in vertebrates, where there are four different Ca_v_β subunits, (β_1_, β_2_, β_3_, β_4_), which are expected to regulate seven pore-forming α_1_ subunits from the two high voltage-activated classes of calcium channels, Ca_v_1.1–Ca_v_1.4 (L-type) and Ca_v_2.1–Ca_v_2.3 (non-L-type) [13]. There are also low voltage-activated calcium channels, Ca_v_3.1–Ca_v_3.3 (T-type), but there is not strong evidence for their regulation by Ca_v_β subunits [7].

The pond snail *Lymnaea stagnalis*, possess a simple set of calcium channels: a single L-type channel gene (LCa_v_1) [14], [15], a single, synaptic, non-L-type channel (LCa_v_2) [16], [17], a T-type channel (LCa_v_3) [18],[19] and a single beta subunit, LCa_v_β [20]. We use this simple model to address what are the core conserved features for β subunit regulation of calcium channels, and to determine what features are adaptive and unique to vertebrates.

We examine the common alternative splicing patterns in the N-terminus and HOOK domains between snail LCa_v_β and vertebrates Ca_v_β subunits. mRNA expression suggests a tissue specificity to the expression of N-terminal isoforms, with LCa_v_β_A_ (eg. structurally resembling vertebrate β1 isoform in sequence) possessing a more generalized expression, while the LCa_v_β_B_ (in exon position of the shorter vertebrate β3 isoforms) has a more discrete, brain specific expression pattern. Molluscs possess a common site of alternative splicing in the HOOK domain, which generates a variably sized exon 7 by use of alternative acceptor site, and is in the same location as mutually-exclusive spliced isoforms (exon 7a,7b, or 7c) [8], [9] in vertebrate Ca_v_β subunits. Ca_v_β subunits in invertebrates (eg. schistosomes [21], snails [20], squid [22] and honeybees [23]) commonly slow the inactivation kinetics of Cav2 channels in a manner like vertebrate β2a subunit [24]–[26], but without the canonical N-terminal palmitoylation site that primarily influences its slow inactivation. We show that the HOOK domain (- isoform) more than the N-terminus (A isoform) of snail LCa_v_β contributes to the slowing of inactivation kinetics of Ca_v_2 channels. The snail Ca_v_β subunits can also slow the inactivation of snail LCa_v_3 T-type channels, but this may be an observable phenomenon *in vitro*, because there is little evidence for Ca_v_β subunits associating with T-type channels in native cells [7]. And lastly, we examine that the capacity that Ca_v_β subunits to promote an increase in the inactivation rate of α subunits while the channels are largely in the closed state [27], [28]. This property known as “closed-state inactivation” is not observable for the snail LCa_v_β or synaptic LCa_v_2 channel, which suggests that this property is more a specialization for specific vertebrate subunit combinations such as Ca_v_β_3_ and Ca_v_2.2 [27], [28].

## Results and Discussion

### 1. Conserved SH3-GK core of Ca_v_β subunits

**Figure 1:**
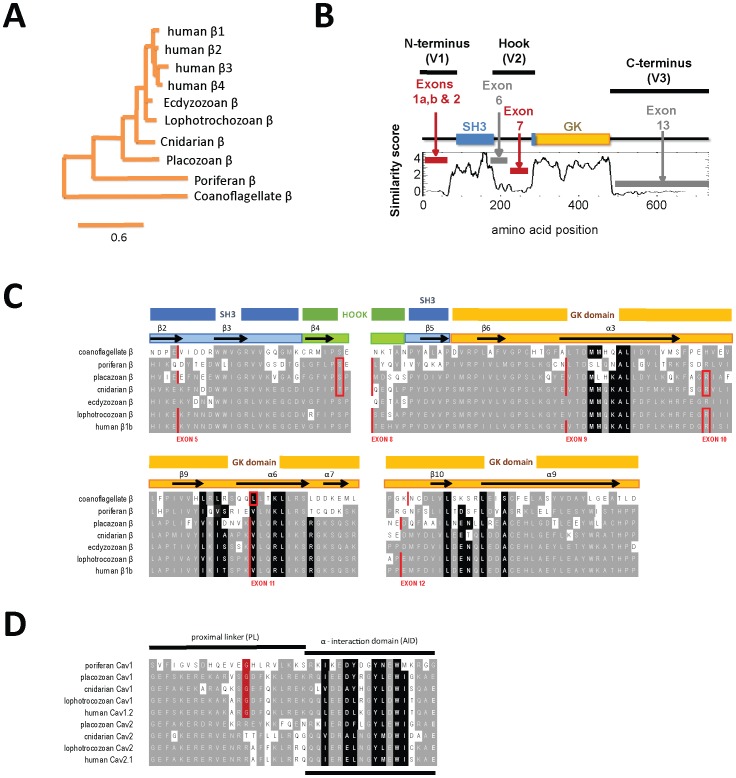
Conservation of calcium channel beta (Ca_v_β) subunits from single-celled organisms to humans. (**A**) Gene tree derived from aligned sequences illustrating the relationship of the four vertebrate Ca_v_β subunits (β1, β2, β3, β4) with the single Ca_v_β representatives in non-vertebrates, including single-celled organisms. (**B**) Running average of similarity (window = 50 amino acids) of aligned invertebrate and human Ca_v_β subunits sequences (in Figure S1). Ca_v_β subunits have highly conserved SH3 and guanylate kinase (GK) domains, with variability in the N-terminus (V1), HOOK domain (V2) and C-terminus (V3). Alternative splicing shared in Ca_v_β subunits in Exons 1a/1b and exon 2 (N-terminus), and exon 7 (HOOK domain). (**C**) Multiple alignment of Ca_v_β subunits from single cell organisms to humans illustrating highly conserved SH3 and GK domains, conserved secondary structures (α helices and Ca_v_β sheets), and calcium channel (AID) binding residues (blackened residues) reported in crystal structures of Ca_v_β subunits [22]–[24]. Exon boundaries are indicated in red. (**D**) Alignment of cytoplasmic region post trans-membrane segment 6 in Domain I of Ca_v_1 and Ca_v_2 calcium channels which forms an expected α helix. Regions include a proximal linker (PL) and the Alpha_1_-Interaction Domain (AID) associated with binding Ca_v_β subunits (blackened residues).

β subunits of calcium channels (Ca_v_β) contain four major representatives in vertebrates (β_1_ to β_4_), represented by a single gene in invertebrates (**Figure 1A**). Ca_v_β subunits are unique members within the MAGUK (membrane associated guanylate kinase) family of proteins [11], [29], [30], which share a structural core consisting of an SH3 domain and a guanylate kinase (GK) domain, separate by a variable HOOK region (**Figure. 1B, 1C**). MAGUKs, are noted for their role as scaffolding and cytoskeletal-organizing proteins, such as post-synaptic density protein (PSD-95), where the SH3 and GK domains form potential intramolecular or intermolecular interactions, with the same or differing MAGUKs, with neither the SH3 nor GK domains have active sites for standard polyproline interactions or nucleotide kinase activity respectively [11], [29], [30]. β subunits of calcium channels lack PDZ domains of standard MAGUKs, but does have a HOOK domain which splits the SH3 domain [31]–[33] (**Figure 1B, 1C**).

The alpha helix at the end of hydrophobic segment 6 in Domain I of Ca_v_1 and Ca_v_2 α_1_ subunits, is largely expected to extend to an alpha1-interaction domain (AID) of conserved amino acids (**blackened amino acids,** **Figure 1D**), which in a crystal structure embeds into a deep hydrophilic groove of the Ca_v_β subunit formed by the guanylate kinase domain [31]–[33] (**blackened amino acids,** **Figure 1C**). The SH3 and GK domains of Ca_v_β subunits (**Figure 1B**), and most notably the key-in-keyhole interaction residues, between the AID sequences of calcium channel α1 subunits and a conserved binding pocket formed by the Ca_v_β subunit GK domain, respectively, are largely conserved down to the simplest known organisms that have calcium channels, the unicellular eukaryotes choanoflagellates (**Figure 1C, 1D**). The most basal unicellular organisms (protozoan) or multicellular animals (sponge, poriferans) have a single Ca_v_1 L-type calcium channel homolog (**Figure 1A**). These Ca_v_1 homologs, in the simplest organisms, are structural predecessors to the high voltage-activated, dihydropyridine-sensitive LCa_v_1 homolog expressed in the pond snail, *Lymnaea stagnalis* [14], [15]. A second and third class of calcium channels likely derived from L-type calcium channels (in ancestral relatives of placozoans, cnidarians) include the non-L-type, Ca_v_2 channel class, noted for their unique roles in mediating synaptic transmission [16], [17], and the low voltage-activated T-type, Ca_v_3 channel class [18], [19]. All Ca_v_1 and Ca_v_2 channels from simple representatives (placozoan, cnidarians) have a hallmark AID sequence [34] for associating with Ca_v_β subunits, which enables a promiscuity and interchangeability of Ca_v_β subunits interacting with differing Ca_v_1 and Ca_v_2 α_1_ subunit classes.

### 2. Variable regions are associated with common alternative splicing

**Figure 2:**
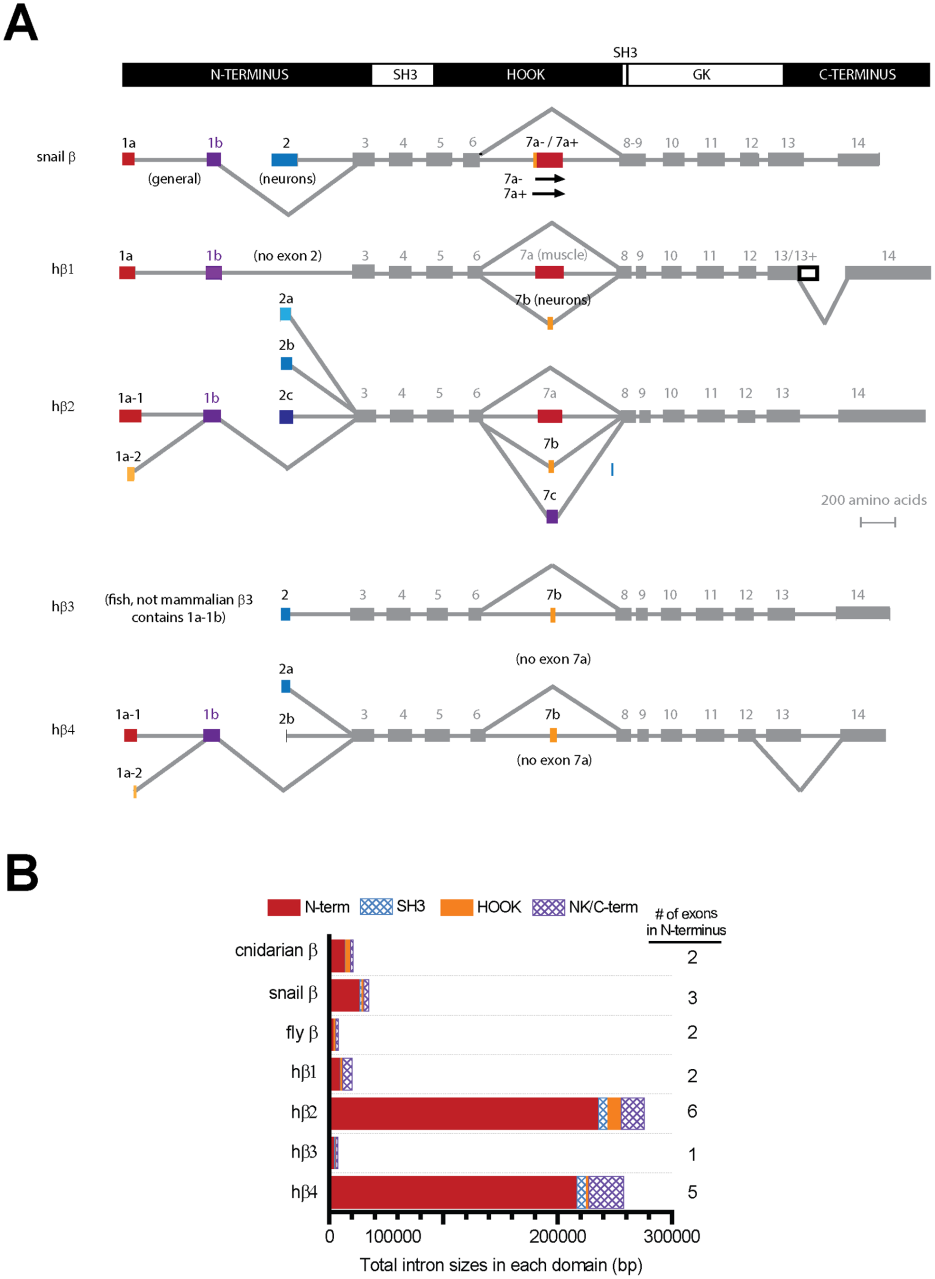
Conserved exon-intron organization, alternative splicing, and N-terminal intron sizes in the genomic sequence spanning calcium channel beta (Ca_v_β) subunits. (**A**) Alignment of the 15 exons of Ca_v_β subunits comparing snail and human Ca_v_β subunit splicing. Ca_v_β subunits have mutually exclusive splicing of N-terminal exon 1a/1b or exon 2 isoforms. Exon 7 in the HOOK domain is subject to mutually-exclusive exon splicing (exon 7a or exon 7b or exon 7c) in vertebrates or splicing in mollusks generated by alternative acceptor sites (exon 7a+, exon 7a−). Molluscan and vertebrate have truncated forms of Ca_v_β subunits lacking the GK domain and C-terminus as a result of skipping of exon 7. (**B**) Most of the intron sizes of Ca_v_β subunits span the N-terminal exons, and the size of the total intron sequence in the N-terminus increases with the number of exons in the N-termini.

Variable regions outside of the conserved SH3 and GK domains of Ca_v_β subunits provide specificity and unique modulation of of different Ca_v_1 and Ca_v_2 channel types [8], [9], [13]. Ca_v_β subunits dramatically vary in the N-terminus (V1), HOOK region (V2) and C-terminus (V3) which is illustrated in a running similarity score (**Figure 1B**) of aligned sequences of invertebrate and mammalian Ca_v_β subunits (**Figure S1**). Alternative splicing of Ca_v_β subunits coincides with these variable regions, illustrated in the exon-intron structure of snail LCa_v_β and the four human Ca_v_β isoforms (shown in **Figure 2A**).

The major pattern of alternative splicing in the four vertebrate Ca_v_β subunits is represented in the molluscan LCa_v_β subunit (as shown in **Figure 2A**), suggesting that the gene splicing patterns evolved in the common ancestor of mollusks and vertebrates, before the duplication that lead to the generation of the our vertebrate isoforms.

### 3. Conservation of N-terminal mutually-exclusive splicing in Ca_v_β subunits (exons 1a/1b and exon 2) and their consistent large intron sizes

**Figure 3:**
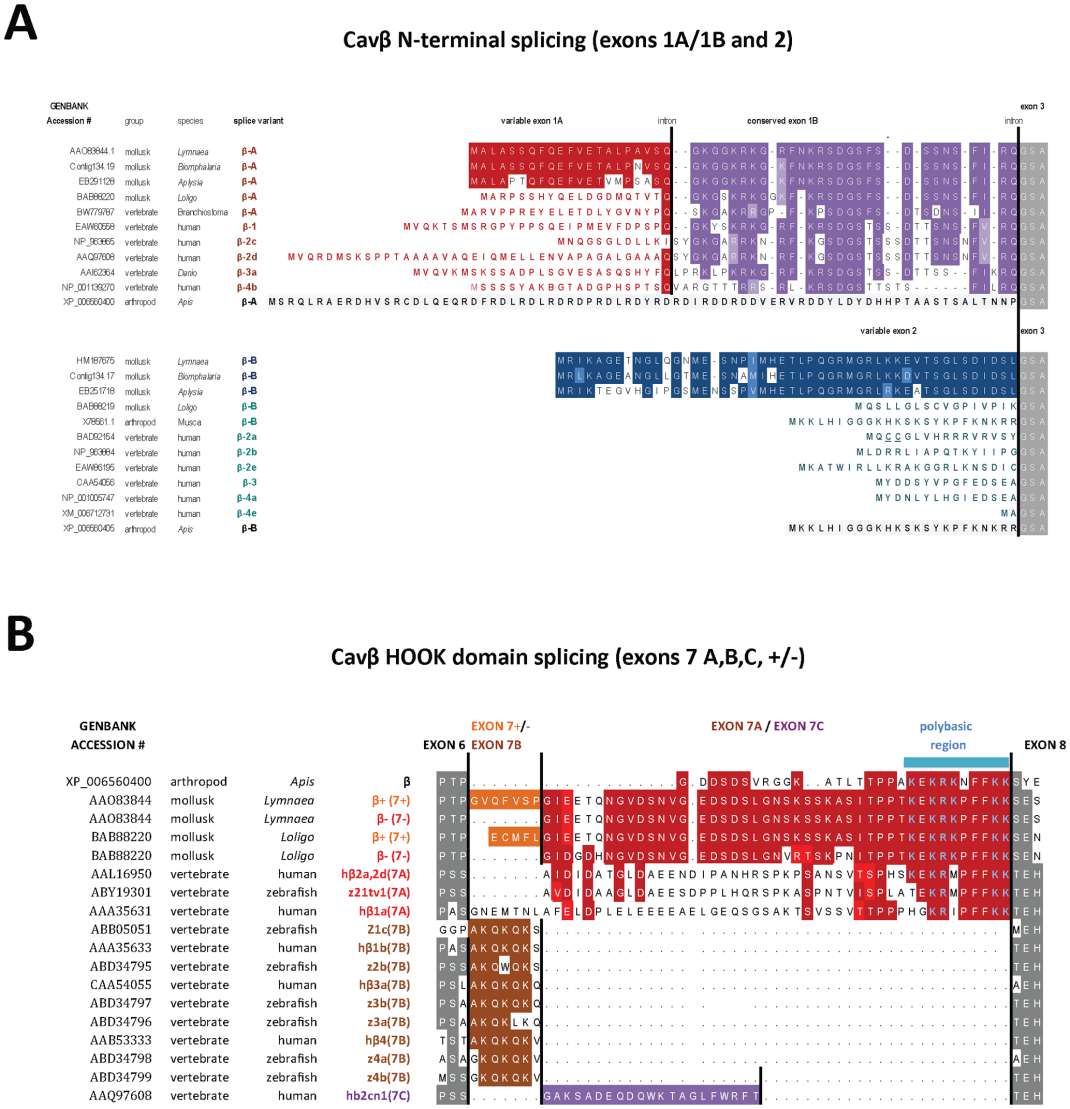
Multiple sequence alignments illustrate conserved splicing in (A) the N-terminus and (B) HOOK domain of invertebrates and vertebrate Ca_v_β subunits. Alternate N-terminal isoforms composed of exons 1a–1b (Ca_v_β_A_) or exon 2 (Ca_v_β_B_), and HOOK domain splicing includes optional short addendum to exon 7 (Ca_v_β−/Ca_v_β+) in invertebrates or mutually exclusive splicing, exon 7a, 7b, 7c. Note that Ca_v_β from snails, squid, schistosomes and bees and vertebrate Ca_v_β_2a_ have slow inactivation kinetics and (**B**) possess HOOK domains with a long form of exon 7 (A form) with a common polybasic region at its 3′ end.

The two most upstream Ca_v_β subunit exons form one N-terminal splice variant, dubbed variant Ca_v_β_A_ (**Figure 3A**), consisting of exon 1a with highly variable sequence, jointed to exon 2b which contains a common conserved amino acid clusters (KxSDSG…FIRQ) shared between molluscan Ca_v_β_A_ and vertebrate spliced isoforms β1, β2c, β2d, β4b. The mutually exclusive N-terminal splice variant Ca_v_β_B_ is formed by exon 2 (**Figure 3B**) and is downstream of exon 1a and exon 1b in the genomic structure of Ca_v_β subunits (**Figure 2A**). Ca_v_β_B_ generates short N-terminal vertebrate isoforms β2b, β2e, β3, β4a including the canonical β2a which has doublet cysteines that are palmitoylated and confer slow inactivation to vertebrate Ca_v_1 and Ca_v_2 calcium channels [24]–[26], and to molluscan LCa_v_2 channels [20]. Honeybee, as a representative insect and arthropod, has two N-terminal spliced isoforms [23], but does not possess a conserved exon 1B with the (KxSDGS…FIRQ) cluster found in molluscan [22] or vertebrate [8], [9] Ca_v_β_B_ isoforms (**Figure 3**).

A general feature of N-terminal exons 1a, 1b and 2 is that the intervening non-coding sequences (introns) that span the region are so large that they compose the majority of the genomic region spanning most Ca_v_β subunits even in a simple cnidarian species, *Nematostella* (**Figure 2B**). The intron sizes are largest in vertebrates isoforms (such as fish and mammalian subunits [35], [36]) which also have the largest number of exon variants that code for exons 1a, and exon 2 in the N-terminus, which are 6 and 4 exons respectively, in vertebrate Ca_v_β2 and Ca_v_β4 subunits (**Figure 2A, 2B**). The genomic region spanning the N-termini for hCa_v_β_4_ and hCa_v_β_2_.is 217,000 bp and 236,000 bp. Gargantuan introns such as in the N-termini of hCa_v_β_4_ and hCa_v_β_2_ are highly unique, and represent ∼0.1% of all introns in the human genome [37]. The evolutionarily conserved, larger sized introns are expected to contain DNA elements which regulate the timing and tissue specificity of the expression of Ca_v_β subunit isoforms.

### 4. mRNA expression confirms a tissue specific expression pattern for N-terminal A and B isoforms

**Figure 4:**
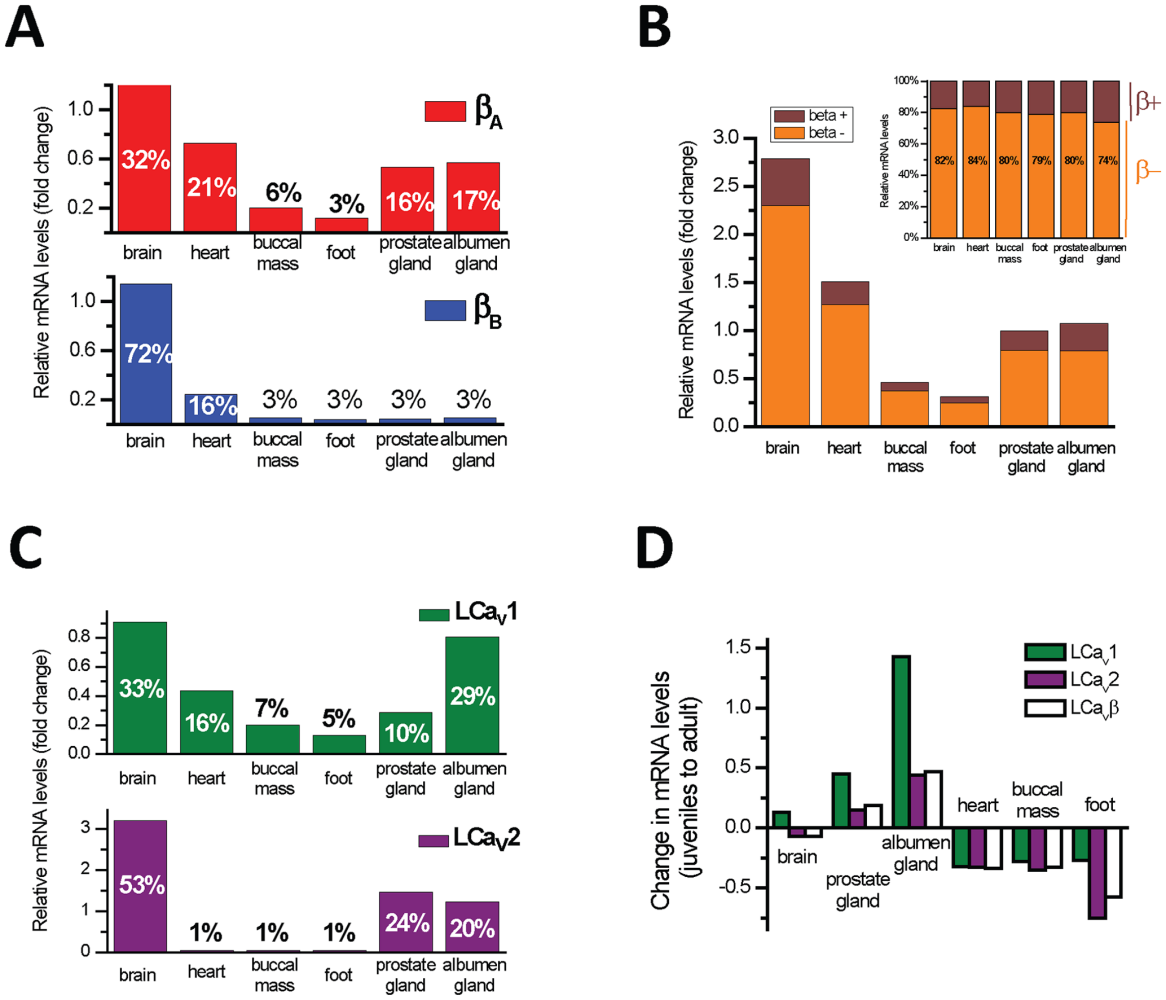
Quantitative RT-PCR results show that N-terminal splice isoforms (LCavβ_A_ and LCavβ_B_), but not HOOK domain splice isoforms (LCavβ− and LCavβ+) of snail Cavβ subunits have tissue specific mRNA expression patterns. mRNA levels are illustrated as fold change relative to HPRT mRNA levels. (A) More generalized pattern of splicing of LCavβ_A_ containing exons 1a/1b, than LCavβ_B_ containing exon 2. Exon 2 containing isoform is mostly expressed in the brain, and residual levels in the heart. (B) Exon 7 splicing generates seven extra amino acids (exon 7a− vs exon 7a+) and appears to have no tissue selectivity pattern of expression. (B, inset) Percent of LCavβ− vs LCavβ+, illustrating that 74%–84% of all transcripts lack the extra amino acids in exon 7. (C) LCa_v_1 L-type channel has a more generalized mRNA expression pattern as LCavβ_A_ while more nervous system specific LCa_v_2 has a more discrete expression pattern as LCavβ_B_ isoforms. (D) Rises and falls in the relative fold changes in mRNA levels from juvenile to adult animals are correlated between LCavβ, LCa_v_1 and LCa_v_2 channel subunits.

mRNA expression patterns suggest that the N-terminus of LCa_v_β, specifically, exons 1a/1b (dubbed “LCa_v_β_A_”) and exon 2 (LCa_v_β_B_”) are highly regulated in their tissue expression (**Figure 4A**), while the HOOK domain splice variants are not (**Figure 4B**). LCa_v_β_A_ is a widely expressed isoform in most tissues (**Figure 4A** **, top**), and resembles the expression profile of the HOOK domain splice isoforms which lack a tissue specific distribution (**Figure 4B**). In contrast, LCa_v_β_B_ is more discretely expressed than LCa_v_β_A_, as a mostly brain specific isoform (72%), with residual expression levels in the heart (16%) (**Figure 4A** **, bottom**). Comparing the relative mRNA levels of the Ca_v_β (**Figure 4A**) with the corresponding mRNA expression of LCa_v_1 and LCa_v_2 channels in different snail tissues (**Figure 4C**) resembles similarities in expression patterns to mammalian gene spliced isoforms [8], [9]. LCa_v_β_A_ (**Figure 4A**) is the primary Ca_v_β subunit isoform expressed in the equivalent tissue resembling skeletal muscle in snails (buccal mass and foot), where LCa_v_1 channel expression dominates (**Figure 4C**). In contrast, LCa_v_β_B_ and LCa_v_2 channel are almost undetectable in the equivalent tissue of skeletal muscle in snails (**Figure 4A**, **Figure 4C**). The combination of mostly LCa_v_1 and LCa_v_β_A_ in snail muscle is consistent with the exclusive pairing of Ca_v_1.1 and Ca_v_β1a in vertebrate skeletal muscle [38]–[40], where vertebrate Ca_v_β1 bears only N-terminal exons 1a/1b like snail LCa_v_β_A_ (**Figure 2A**). It is also noteworthy that mammalian β_3_ lacks N-terminal exons 1a/1b and is composed of exon 2 only (**Figure 2A**) like the snail LCa_v_β_B_ isoform. Mammalian Ca_v_β_3_ is mostly a brain specific subunit that pairs more often with synaptic Ca_v_2.2 channels [41], , and in this regard, resembles the splice variant of snail LCa_v_β_B_ which is also is more brain specific variant (**Figure 4A**) and composed of exon 2. These are examples of likely common pairings of calcium channel and beta subunit isoform between invertebrates and vertebrates, but outside of this, there appears to be a lot of promiscuity and overlap between Ca_v_1 and Ca_v_2 channels and their association with the differing Ca_v_β subunit isoforms. It is noteworthy that “A” isoforms are found in examples of all four vertebrate Ca_v_β subunits, and only Ca_v_β_3_ is lacking a “B” isoform in vertebrates (**Figure 4C**).

Changes in mRNA levels of Ca_v_β subunits from juvenile to adult snails, match the direction of change of the corresponding mRNA of calcium channels (**Figure 4D**). Heart and muscle mRNA levels fall from juvenile to adult animals for LCa_v_1 and LCa_v_2 channels and LCa_v_β subunits, while there is a rise in LCa_v_1 and LCa_v_2 channels and LCa_v_β subunits corresponding with the maturation of sexual organs from juvenile to adult animals, such as in prostate and albumen gland (**Figure 4D**).

### 5. Common HOOK domain splicing of exon 7 in molluscan and vertebrate Ca_v_β subunits

The HOOK domain is a second area of variability in Ca_v_β subunits and subject to alternative splicing. The HOOK domain is unstructured and not resolvable in the crystal structure of Ca_v_β subunits [31]–[33]. The HOOK domain splits the SH3 domain, separating the 5^th^ Ca_v_β strand from the rest of the SH3 domain (**Figure 1B** **,** **Figure 1C** and **Figure S1**). The variability in the HOOK domain is largely contained in exons 6 and 7, where exon 7 is subject to alternative splicing that is shared in molluscan and mammalian Ca_v_β subunits (**Figure 2A**).

Mutually-exclusive splicing of exon 7 in mammals leads to either a skipping of exon 7 altogether, or includes a choice of one of three exon 7 variants including, exon 7a (a long form: e.g. AIDID…PFFKK), exon 7b (a short form: AKQKQKQ) and more rarely exon 7c (**Figure 3B**) [8], [9]. Snails (and also squid [22]) can also exclude exon 7 altogether, via use of alternative acceptor sites (**Figure 2A**) or generating exon 7 containing seven or five extra amino acids (exon 7+), respectively, or versions lacking these extra amino acids (exon 7−) (**Figure 3B**, **Figure S2**). Invertebrate exon 7 resembles the exon 7a (long form) of vertebrate Ca_v_β subunits, with a conserved polybasic region at its 3′ end (KRxPFFKK) (**Figure 3B**) [43]. The skipping of exon 7 is found in mammalian Ca_v_β subunits [44]–[46] and shared in molluscan Ca_v_β subunits and generates a frame shift and an immediately truncated Ca_v_β subunit that lacks the GK domain and C-terminus (**Figure 2A**). The frame shift occurs because exon 6 ends within a codon after the first nucleotide (phase 1), whereas the intron preceding exon 8 is located between codons (phase 0) (**Figure S2**). One role discovered for the truncated Ca_v_β subunit in vertebrates is that it transports into the nucleus and serve as a transcription factor [47]–[50]. Conservation of splicing that skips exon 7 suggests that truncated Ca_v_β subunits in invertebrates may also have similar roles outside of their association with Ca_v_1 and Ca_v_2 calcium channels.

### 6. HOOK domain spliced variants lack a tissue specific expression pattern

Snail LCa_v_β+ or LCa_v_β− containing or not containing the extension to exon 7, respectively, show no obvious tissue preference in their mRNA expression. Notably LCa_v_β− (lacking the extension to exon 7) is more common, being 74%–84% of the total mRNA transcript in all snail tissues (**Figure 4B**). The lack of tissue regulation for splicing in the HOOK domain contrasts with the more highly regulated N-terminal alternatively-spliced exons. There is also consistently smaller and more normal sizes of introns spanning exon 7, which contrasts with the very large to gargantuan introns spanning the N-terminal exons (**Figure 2B**).

### 7. Invertebrate Ca_v_β subunits slow the inactivation kinetics of Ca_v_2 channels

We have co-expressed full-length isoforms of LCa_v_1, LCa_v_2 and LCa_v_3 channels with mammalian α_2_δ_1_, and the four combinations of LCa_v_β isoforms in HEK-293T cells to assess the consequences of LCa_v_β splice isoforms on the expression of the calcium channels recorded using whole-cell patch clamp electrophysiology. Combinations that we assessed were LCa_v_β with exon1a/1b (**A form**) or exon 2 (**B form**), and LCa_v_β channels with or without the optional seven amino acids in exon 7 (i.e. **+ form** or **− form**, respectively): (LCa_v_β_A_+, LCa_v_β_B_+, LCa_v_β_A_−, LCa_v_β_B_−) compared to the absence of co-expressed Ca_v_β subunit. We substituted native external calcium ions for barium ions to evaluate the consequences of calcium-independent effects on biophysical properties recorded in whole cell voltage clamp, avoiding the dramatic calcium dependent inactivation, characteristic of LCa_v_1 channels, observed when external calcium in the charge carrier. Reported effects of Ca_v_β subunits have focused on the dramatic changes to the voltage-dependent properties [7]–[9], so we have limited our study to an examination of barium currents. All electrophysiology results +/− s.e.m. are tabulated in **Table 1**.

**Table 1:** Summary of electrophysiology parameters for Figures 5–8.

Figure 5 and 7. Voltage-sensitivities of LCa_v_1, LCa_v_2, LCa_v_3 channels with and without LCa_v_β splice isoforms												
	Activation V_0.5_ (mV)		p value	Activation K_a_	n	p value	Inactivation V_0.5_ (mV)	n	p value	Inactivation K_i_	n	p value
LCa_v_1 & no β	3.27±4.27	8		7.83±1.50	8		−20.29±4.56	3		8.30±1.72	3	
LCa_v_1 & LCa_v_β_A_+	2.07±1.05	7	n.s.	7.09±1.21	7	n.s.	−16.47±4.27	3	n.s.	7.56±1.50	3	n.s.
LCa_v_1 & LCa_v_β_B_+	2.47±1.03	24	n.s.	6.77±0.76	24	n.s.	−21.33±2.18	5	n.s.	8.57±1.69	5	n.s.
LCa_v_1 & LCa_v_β_B_−	3.55±1.57	5	n.s.	6.96±1.74	5	n.s.	−18.09±3.72	3	n.s.	8.25±1.45	3	n.s.
LCa_v_2 & no β	12.11±2.20	5		6.43±1.91	5		−31.28±6.76	4		10.90±1.21	4	
LCa_v_2 & LCa_v_β_A_+	12.30±0.96	10	n.s.	6.74±0.27	10	n.s.	−29.18±2.32	7	n.s.	11.02±1.63	7	n.s.
LCa_v_2 & LCa_v_β_B_+	12.07±1.21	8	n.s.	6.49±1.74	8	n.s.	−33.02±3.20	4	n.s.	9.15±2.01	4	n.s.
LCa_v_2 & LCa_v_β_B_−	9.95±1.07	5	*	6.13±0.30	5	n.s.	−32.37±2.57	4	n.s.	9.26±1.21	4	n.s.
LCa_v_3 & no β	−67.65±1.20	9		4.68±0.20	9		−84.12±0.38	3		3.16±0.15	3	
LCa_v_3 & LCa_v_β_A_+	−68.71±2.69	10	n.s.	4.64±0.61	10	n.s.	−84.82±1.19	3	n.s.	3.28±0.24	3	n.s.

Figure 5 and 7. LCa_v_1, LCa_v_2 and LCa_v_3 current densities with and without LCa_v_β splice isoforms						
Figure 6 and 7. Quantification of Inactivation kinetics of LCa_v_1, LCa_v_2 and LCa_v_2 currents with and without LCa_v_β splice isoforms						
	Current density (pA/pF)	n	p value	I (at 250 ms)/I_max_	n	p value
LCa_v_1 & no β	7.55±1.05	8		0.800±0.060	4	
LCa_v_1 & LCa_v_β_A_+	9.36±0.52	7	**	0.736±0.045	7	n.s.
LCa_v_1 & LCa_v_β_A_−	24.36±3.95	7	***	0.747±0.049	7	n.s.
LCa_v_1 & LCa_v_β_B_+	19.43±3.77	24	***	0.730±0.076	6	n.s.
LCa_v_1 & LCa_v_β_B_−	15.03±0.99	5	***	0.727±0.061	5	n.s.
LCa_v_2 & no β	4.80±1.17	5		0.038±0.061	6	
LCa_v_2 & LCa_v_β_A_+	13.49±2.92	10	***	0.299±0.013	12	***
LCa_v_2 & LCa_v_β_A_−	17.43±2.10	4	***	0.239±0.025	4	***
LCa_v_2 & LCa_v_β_B_+	21.95±9.78	8	***	0.282±0.013	6	***
LCa_v_2 & LCa_v_β_B_−	24.36±3.95	7	***	0.229±0.008	6	***

	Current density (pA/pF)	n	P value	Tau at −40 mV (ms)	n	P value
LCa_v_3 & no β	95.43±24.94	10	n.s.	10.94±0.32	5	***
LCa_v_3 & LCa_v_β_A_+	104.96±32.41	11	n.s.	13.14±0.44	6	***

Figure 8. Analyses of closed-state inactivation for Cav2.2 and LCav2 with snail and mammalian beta subunit combinations						
	I_test_/I_pre_ at 0.5 ms	n		I_test_/I_pre_ at 8 ms	n	p value
Ca_v_2.2 & Ca_v_β_3_	0.772±0.009	8		0.707±0.011	8	***
Ca_v_2.2 & Ca_v_β_1b_	0.829±0.025	6		0.823±0.025	6	n.s.
Ca_v_2.2 & Ca_v_β_2a_	0.971±0.036	3		0.978±0.068	3	n.s.
Ca_v_2.2 & LCa_v_β_A_+	0.979±0.010	3		0.969±0.014	3	n.s.
LCa_v_2 & Ca_v_β_3_	0.647±0.034	7		0.821±0.030	7	***
LCa_v_2 & Ca_v_β_1b_	0.715±0.095	3		0.853±0.047	3	***
LCa_v_2 & Ca_v_β_2a_	1.008±0.085	5		1.023±0.031	5	n.s.
LCa_v_2 & LCa_v_β_A_+	0.917±0.059	5		0.976±0.095	5	n.s.

**Figure 5:**
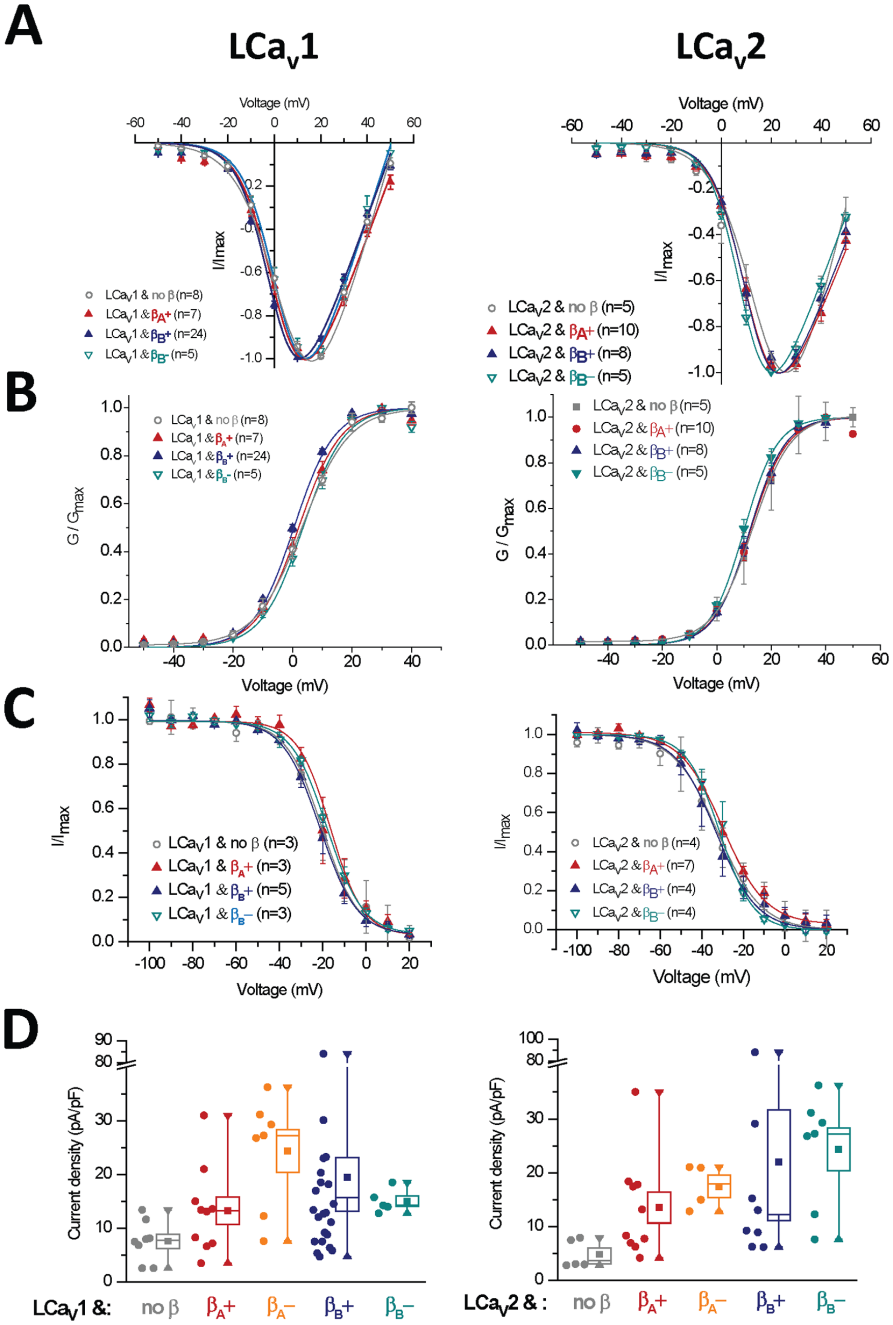
Snail beta subunit (LCa_v_β) splice isoforms containing either N-terminal exons 1a/1b or exon 2 (A or B) or insert in exon 7 (7a− vs 7a+), boost the membrane expression of snail LCa_v_1 or LCa_v_2 channels but has no effect on their voltage-sensitivities when external barium is the charge carrier. (**A**) Current-voltage relationships (curve-fitted with Ohmic-Boltzmann equation) (**B**) Activation and (**C**) Steady-state inactivation curves (curve-fitted with Boltzmann equation). (**D**) Current densities (pA/pF) shown as box plot (box = s.e.m., whisker range = minimum/maximum values).

**Figure 6:**
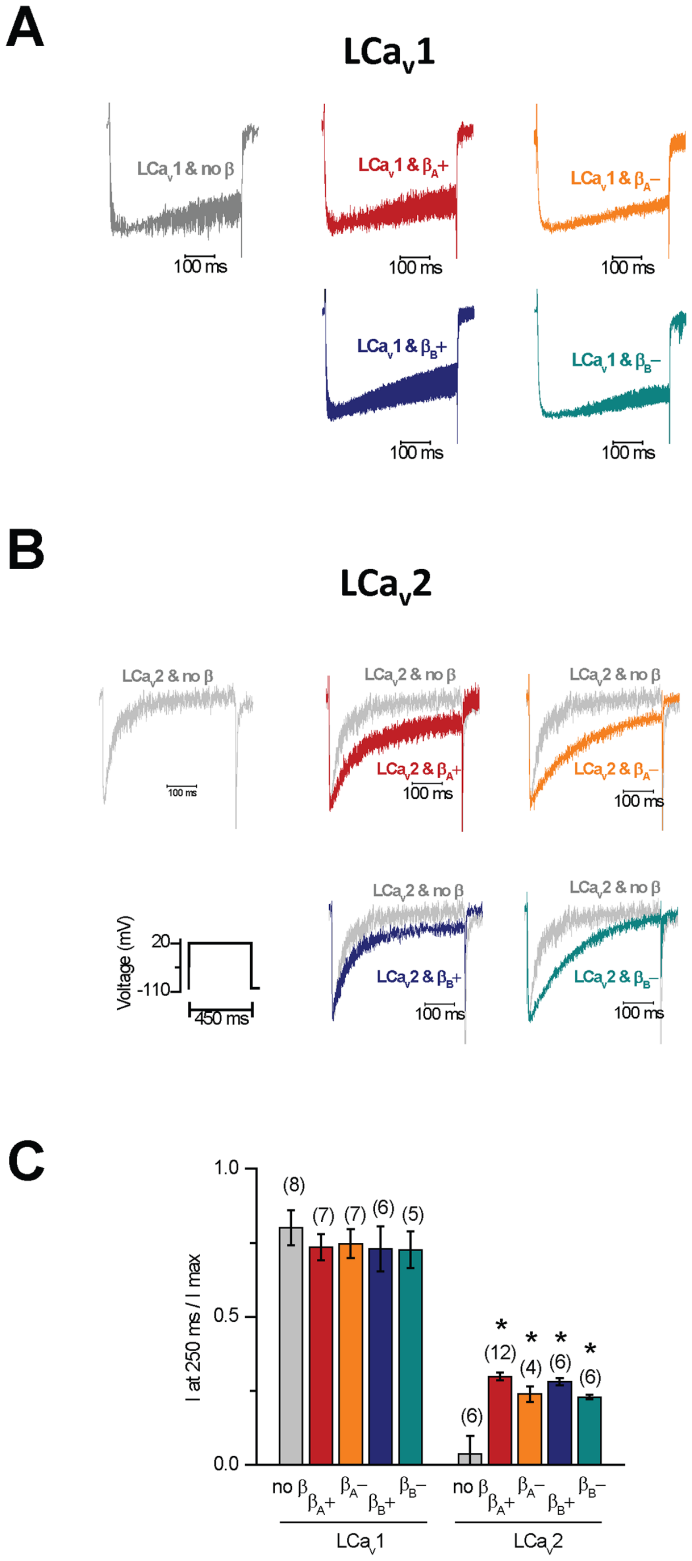
Snail beta subunit (LCavβ) splice isoforms containing either N-terminal exons 1a/1b or exon 2 (A or B) or insert in exon 7 (7a− vs 7a+), slow the inactivation kinetics of LCa_v_2 channels. Four representative and normalized peak barium current traces shown as mean, s.e.m. for (A) LCa_v_1 and (B) LCa_v_2 channels co-expressed with LCavβ splice isoforms or no Cavβ. (C) Rate of inactivation decay reflected in the fraction of maximal peak current at 250 ms time point. All LCavβ splice isoforms like vertebrate Cavβ_2a_ slow inactivation kinetics of LCa_v_2 channels. The slowing of inactivation kinetics is maximized with the exon 7a− configuration.

We found that none of the snail LCa_v_β isoforms influenced the current voltage-relationships such as the threshold voltage at which barium currents are beginning to be visible or the voltage of peak current for LCa_v_1 or LCa_v_2 channels (**Figure 5A**). This absence of a difference is reflected in the activation curves fitted with a Boltzmann equation (**Figure 5B**). Similarly there were also no effects of LCa_v_β subunits on steady-state inactivation (**Figure 5C**). This is different from the dramatic effects of mammalian Ca_v_β subunits on mammalian Ca_v_1 and Ca_v_2 channels [7]–[9]. Mammalian Ca_v_β subunits impart large shifts in the voltage-dependence of activation to more hyperpolarized voltages by approximately −10 mV to −20 mV [7], [8]. Mammalian Ca_v_β subunits, except for Ca_v_β_2a_ also cause an approximately 10 mV hyperpolarizing shift in steady-state inactivation curves, and accelerate inactivation kinetics to varying degrees [7]–[9]. Ca_v_β_2a_ has doublet cysteines that are palmitoylated in an exon 2 type N-terminus, which promote membrane association of Ca_v_β subunits [24]–[26]. Palmitoylation is considered to anchor and immobilize Ca_v_β_2a_, causing calcium channels to be more reluctant to inactivate (shifting inactivation curves in the depolarizing direction), and is responsible for much of the slowing of its inactivation kinetics [24]–[26]. Snail LCa_v_β resembles Ca_v_β_2a_ in promoting the slowing of inactivation kinetics of LCa_v_2 channels (**Figure 6B, 6C**), albeit to a lesser degree than the slowing of inactivation kinetics of LCa_v_2 induced by Ca_v_β_2a_ (see figure 3e in [20]). But notably, the slowing of inactivation kinetics is a consistent feature in other invertebrate Ca_v_β subunits (such as schistosome [21], squid [22] and honeybee [23] Ca_v_β subunits), and these subunits, including snail, lack N-terminal palymitoylation residues in the N-termini of their Ca_v_β subunits.

### 8. The HOOK domain more than the N-terminus contributes to the slowing of the inactivation kinetics imparted by invertebrate Ca_v_β subunits

We observe that splicing in the HOOK domain of snail LCa_v_β− lacking extra residues in exon 7, has a more dramatic effect on the slowing of the inactivation kinetics of LCa_v_2 channels compared to snail LCa_v_β+ possessing extra residues in exon 7 (**Figure 6B, 6C**). In contrast, the differing N-termini of LCa_v_β do not influence the inactivation kinetics of LCa_v_2 (**Figure 6B, 6C**). We observed no measureable electrophysiological differences between the expression of LCa_v_β_A_ (exon 1a/1b) and LCa_v_β_B_ (exon 2) isoforms, suggesting that the N-terminal splicing is more associated with tissue localization than to altering biophysical properties of snail Ca_v_1 and Ca_v_2 channels. Size of the N-terminus may also be a relevant parameter for LCa_v_β_A_ and LCa_v_β_B_, since these variants have similarly sized N-termini (50 vs. 47 amino acids respectively). Differing lengths of N-termini of mammalian Ca_v_β_1b_ [51] and Ca_v_β_2_ [52] channels are known to correlate with altering rates of inactivation in mammalian Ca_v_2 channels.

Comparing different invertebrate Ca_v_β subunits (schistosome [21], snail [20], squid [22] and honeybee [23]), the N-termini are highly variable in sequence, yet all impart slow inactivation kinetics of Ca_v_2 channels in the absence of palmitoylation residues found in vertebrate Ca_v_β_2a_ Likely more relevant than the N-terminus for imparting slow inactivation kinetics is the HOOK domain which in invertebrate Ca_v_β subunits resembles Ca_v_β_2a_ (longer spliced isoform, Exon 7A) with a conserved polybasic residue stretch at the 3′ end [43], [53]. The polybasic residue stretch is lacking in vertebrate β_1b_, β_3_ and β_4_ subunits, containing the shorter spliced isoform (Exon 7B instead of Exon 7A). These other channel types (Ca_v_β_1b_, Ca_v_β_3_ and Ca_v_β_4_) bear faster inactivation kinetics than Ca_v_β_2a_ [8], [9]. The conserved polybasic residue stretch in Exon 7A contributes to the slow inactivation kinetic phenotype, even in the absence of palmitoylation residues in the N-terminus found in vertebrate Ca_v_β_2a_ channels [43], [53]. The simplest evolutionary hypothesis is that vertebrate Ca_v_β_2a_ retained the extended Exon 7A with the polybasic region in the HOOK domain shared in a common ancestral Ca_v_β subunit (resembling the invertebrate Ca_v_β subunit) to complement its very slow inactivation phenotype governed largely by its unique N-terminus of palmitoylation residues. Every invertebrate Ca_v_β subunit (eg. cnidarian, nematode, schistosome, mollusk, insect) resembles Exon 7A with the polybasic region in the HOOK domain, which may suggest that the shorter HOOK domain of Exon 7B was a vertebrate adaptation to promote a faster inactivation phenotype for Ca_v_β_1b_, Ca_v_β_3_ and Ca_v_β_4_ subunits.

### 9. Invertebrate Ca_v_β subunits do not alter the inactivation kinetics of LCa_v_1 L-type channels

Snail LCa_v_1 channels possess little voltage-dependent inactivation without Ca_v_β subunit expression, so it is not surprising that we did not observe additional slowing of inactivation kinetics imparted by LCa_v_β on LCa_v_1 channels when barium is the external charge carrier (**Figure 6A, 6C**). Snail LCa_v_1 channels have a prominent calcium dependent inactivation [14], [54] as mammalian Ca_v_1.2 channels [55], which has been attributabed to a conserved C-terminal IQ motif and a N-terminal NSCATE motif for binding calmodulin in the presence of external calcium [14]. The much faster voltage-dependent inactivation of vertebrate Ca_v_2 channels has been attributed to the more helical, and rigid proximal linker (PL) between the inactivation gate of the transmembrane segment 6 (S6) helix of domain I and the AID sequence for Ca_v_β subunit binding [56]–[58]. Both invertebrate and vertebrate Ca_v_1 channels have a conserved glycine in the proximal linker (**Figure 1D**) which is expected to lower the alpha-helicity and rigidity of the helix, and weaken the observed influences of the Ca_v_β subunit on the inactivation kinetics of Ca_v_1 channels [57]. The conservation of mammalian calcium channel features in the most primitive multicellular animals is consistent with the notion that a common ancestor before the appearance of nervous systems (i.e. resembling an extant placozoan), possessed a Cav2 channel homolog with a strongly voltage-dependent regulation by Ca_v_β subunits, and a Cav1 channel homolog with a calcium-dependent regulation of inactivation kinetics by calmodulin.

### 10. Invertebrate Ca_v_β subunits slow the inactivation kinetics of Ca_v_3 channels *in vitro*

**Figure 7:**
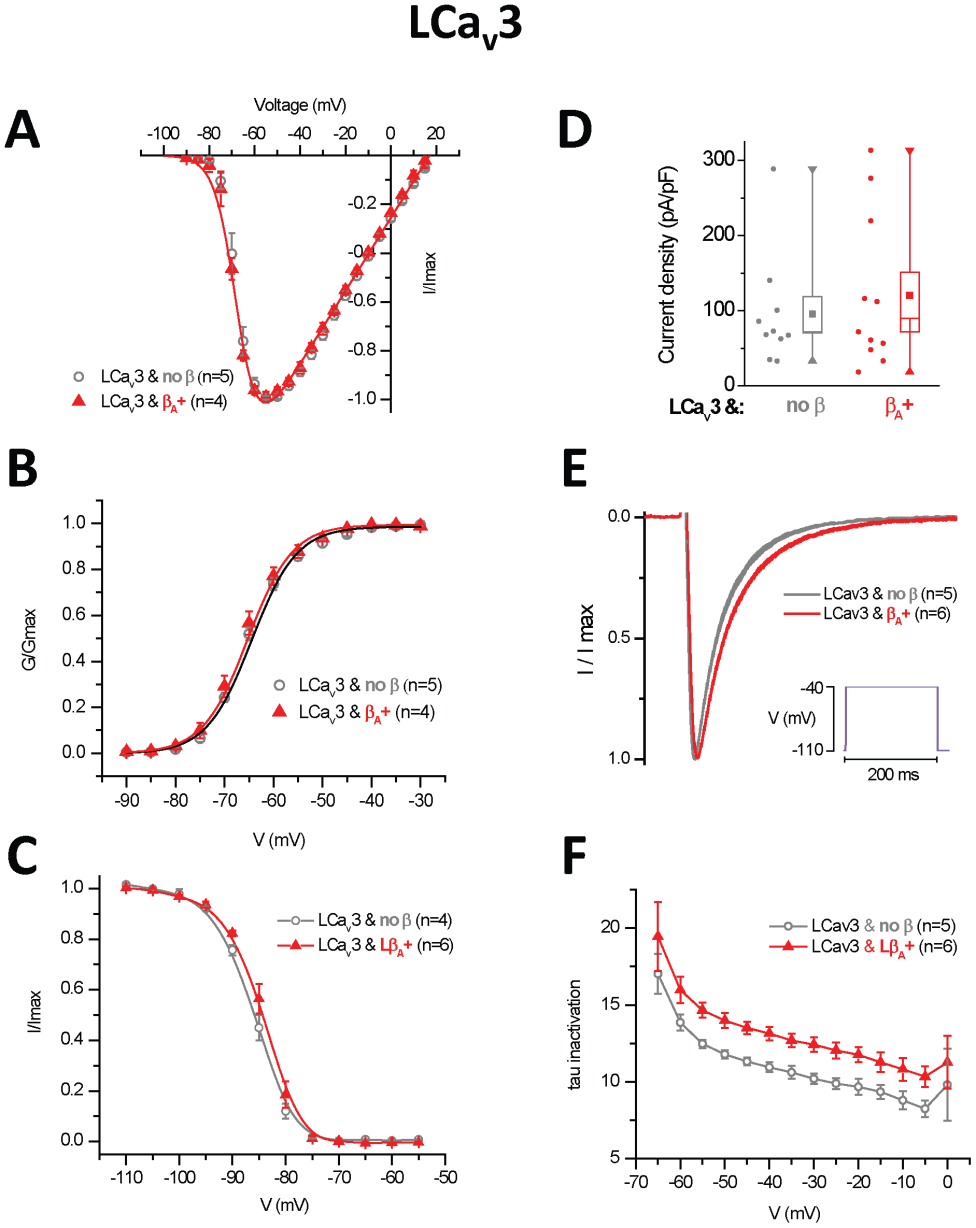
Snail beta subunit (LCavβ_A_+) splice isoform slow the inactivation kinetics, but has no effect on any other biophysical property on snail T-type, LCa_v_3 channels. (A) Current-voltage relationships (B) Activation and (C) Steady-state inactivation curves. (D) Current densities (pA/pF) shown as box plot (box = s.e.m., whisker range = minimum/maximum values). (E) Normalized peak barium current traces illustrated as mean, s.e.m. for LCa_v_3 channels co-expressed with LCavβ splice isoforms or no Cavβ. (F) Curve fitting of inactivation (Tau values) over the steps from −110 mV to the range of −70 mV to 0 mV).

There have been reports that Ca_v_3 T-Type channels can be modulated by the co-expression of Ca_v_β subunits *in vitro* [59]–[61]. We report that the snail Ca_v_β can promote the slowing of inactivation kinetics of LCa_v_3 channels in a manner similar to LCa_v_2 channels (**Figure 7E,F**), and this modulation occurs without any effect on other biophysical properties (**Figure 7A,B,C**) or membrane expression levels (**Figure 7E**). This commonly observed phenomenon could be an artifact of *in vitro* studies, since there isn't strong evidence that Ca_v_3 channels are modulated with Ca_v_β subunits *in vivo*. Reported β subunit interactions are weak with T-type channels [59]–[61], and require the transfer of I–II linker sequences from Cav1 or Cav2 channels to acquire characteristic features of Ca_v_β subunit modulation [62]. Also, native Ca_v_3-Ca_v_β protein complexes have never been identified, knockdown of Ca_v_β subunits in native cells do not alter T-type channel properties [63], [64], and snail LCa_v_3 [18], [19] and mammalian [65] T-type channels are easily reconstituted and resemble the features of native currents *in vitro*, without having to co-express Ca_v_β subunits.

### 11. A lack of closed state inactivation in the Ca_v_β subunit of invertebrates

**Figure 8:**
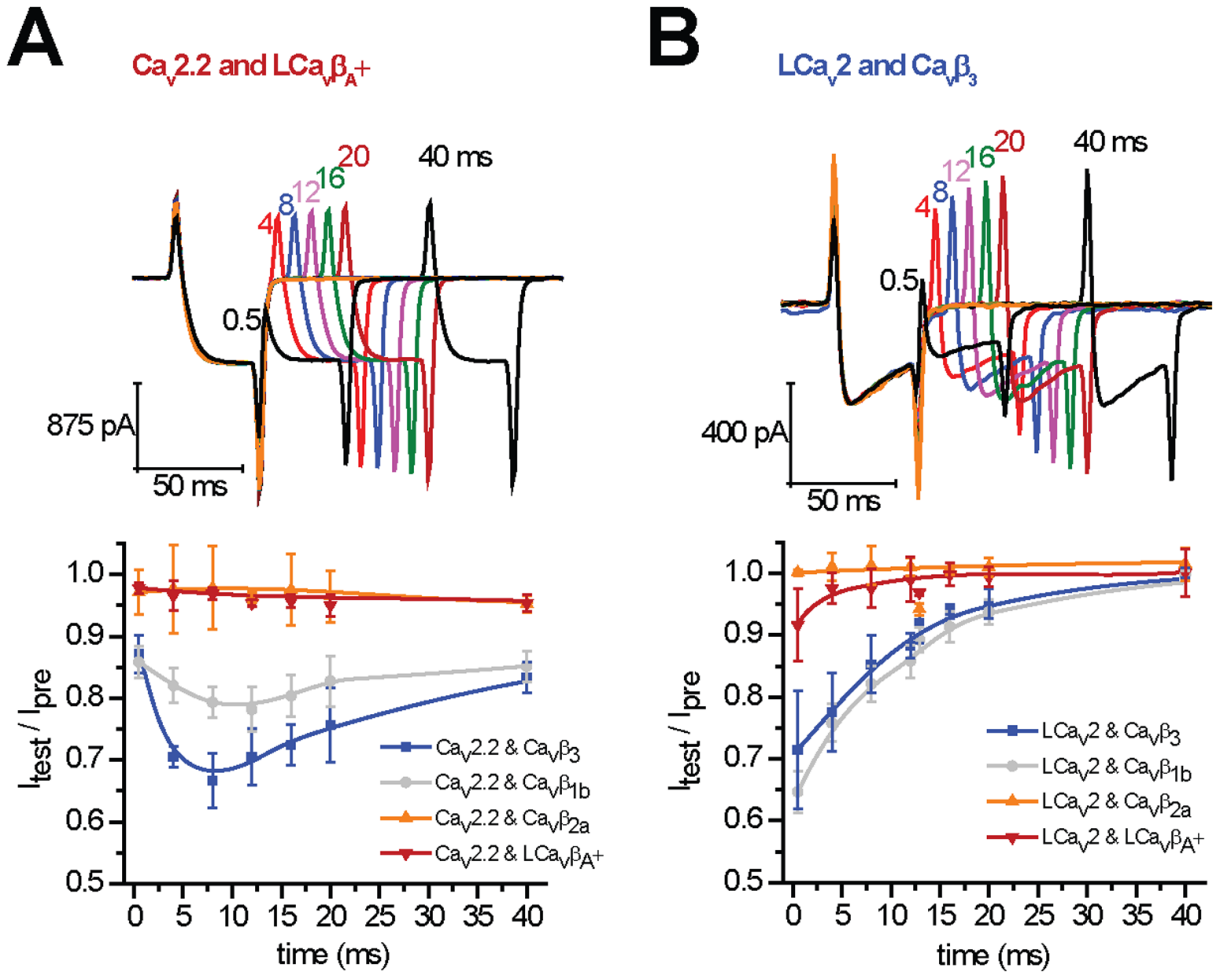
Snail Ca_v_2 channels nor snail LCa_v_β subunits do not promote the closed-state inactivation observed for mammalian Ca_v_2.2 and Ca_v_β_3_ or Ca_v_β_1b_ subunits. The size of test barium currents relative to the prepulse current after time delays of 0.5, 4, 8, 12, 16, 20 and 40_v_β_3_, Ca_v_β_1b_, Ca_v_β_2a_ and snail LCa_v_β_A_+ with mammalian Ca_v_2.2 or snail LCa_v_2 calcium channels. A closed-state inactivation exhibited where there is an increasing inactivation with increasing time delay, is found only with particular combination of subunits, which includes mammalian Ca_v_2.2 and Ca_v_β_3_ or Ca_v_β_1b_ [18], [19].

Another feature that has been attributed to Ca_v_β is the tendency of Ca_v_2 channels to inactivate more than expected during a train of action potentials [27], [28]. This is a feature dubbed “closed-state inactivation”, where Ca_v_β subunits promote a greater inactivation during the repolarization period, when Ca_v_2 channels are expected to be more unavailable, in non - open and inactivated states [27], [28]. Observation of this persistent inactivation can be obtained by using a two-pulse protocol, where a prepulse step depolarization is applied to generate maximal currents, and then the percent of recovery of the same peak currents assessed by a second voltage step after a time delay (e.g. 0.5 to 40 ms) [28]. With a short time delay between voltage pulses, such as 8 ms, there is a “dip” in the time of recovery observed for mammalian Ca_v_2.2 channels co-expressed with Ca_v_β_3_, in particular where a greater than expected fraction of Ca_v_2.2 channels are observed to be unavailable for opening compared to more brief time periods (0.5 ms) or longer time periods (40 ms) of inactivation recovery between the prepulse and the test pulse (**Figure 8A** **, bottom panel**). We observe that the closed-state inactivation is a property that is most evident for Ca_v_β_3_ and less for Ca_v_β_1b_, and not observable for Ca_v_β_2a_ (**Figure 8A** **, bottom panel**), as has been reported previously [28]. Snail LCa_v_β_A_+ is similar to Ca_v_β_2a_ in lacking closed state inactivation (**Figure 8** **, sample trace shown in upper panel**). Not only was the snail Ca_v_β unable to bestow a closed state inactivation on mammalian Ca_v_2.2 channels, but the snail LCa_v_2 channel was not compatible with closed state inactivation either. None of the Ca_v_β subunits including LCa_v_β_A_+ or mammalian Ca_v_β_3_ or Ca_v_β_2a_ could generate the closed state inactivation channel behavior on LCa_v_2 (**Figure 8B**).

### 12. Calcium current density changes due to Ca_v_β subunit expression

Ca_v_β subunits are required for expression of Ca_v_1 and Ca_v_2 channels and little or no surface expression *in vitro* and *in vivo* is observed in their absence. Binding of Ca_v_β subunits to calcium channels in the Alpha1 Interaction Domain (AID) sequence of the linker is modeled to promote membrane trafficking and expression of calcium channels, by facilitating their export from the endoplasmic reticulum [66] and preventing ubiquitination and proteosomal degradation [67]–[69]. Previously we were unable to measure an increase in current density of expressed snail LCa_v_2 channels with co-expressed snail LCa_v_β, when LCa_v_2 channels contained an N-terminus of Ca_v_2.2 [20]. We show here that expression of both LCa_v_1 and LCa_v_2 with its native N-terminus produced statistically significant increases in current density with the expression of all snail LCa_v_β subunit isoforms (**boxplots in** **Figure 5D** **, and see statistics in** **Table 1**).

### 13. Conclusions

Ca_v_β subunits found in single-celled coanoflagellates to humans, bear highly conserved functional core of SH3 and GK domains for associating and modulate high voltage-gated calcium channels. Variable regions in the N-terminus (exons 1a/1b and 2) and HOOK domain (exon 7) contain conserved alternative splicing that is shared amongst Ca_v_β subunits. Molluscan (squid [22], snail [20]) schistosome [21] and insect (bee) [23] Ca_v_β subunits all substantially slow inactivation kinetics of Ca_v_2 channels despite differing spliced N-termini of exon 1a/1b or exon 2 isoforms. The slowing of inactivation kinetics of invertebrate Ca_v_β subunits occurs in the absence of the canonical palmitoylated residues found the N-terminus of vertebrate Ca_v_β_2a_ subunit, but notably the slowing of inactivation kinetics correlates with the presence of a stretch of polybasic residues at the 3′ end of the HOOK domain common to vertebrate Ca_v_β_1_ and Ca_v_β_2a_ subunits. While alternative splicing of the HOOK domain is primarily responsible for promoting the slowing of inactivation of invertebrate Ca_v_β subunits, alterative splicing of the N-terminus relates to a tissue specific expression pattern that is found in Ca_v_β subunits. An N-terminal “A isoform” composed of exon 1a/1b is the predominant form expressed in skeletal muscle (although A isoforms of Ca_v_β subunits are broadly expressed across tissues), while some “B isoforms” can have a highly brain-centric expression pattern (although this is not true of all “B isoforms” of Ca_v_β subunits). Specialization such as closed-state inactivation [18], [19] are features that evolved within vertebrates tailored to specific Ca_v_β isoforms and Ca_v_2 calcium channel types. Further studies in invertebrates will further clarify the nature of the relationship between calcium channels and Ca_v_β, and it is a model where the numbers of possibilities are limited, given that there are only single genes coding for Ca_v_1, Ca_v_2, Ca_v_3 and Ca_v_β in the snail genome.

## Materials and Methods

### Cloning of novel LCa_v_β subunit isoforms

The calcium channel beta subunit originally cloned and expressed from the pond snail *Lymnaea stagnalis* was LCa_v_β_A_+ and previously described [20], and deposited as GenBank Accession # AF484087. Novel LCa_v_β_A_−, LCa_v_β_B_+ and LCa_v_β_B_− splice isoforms have been deposited as HM187674.1, HM187675.1 and HM187676.1 respectively. Novel exons in the N-terminus and exon 7 were first identified by PCR amplification of λZAP cDNA libraries and freshly-isolated genomic DNA. The 5′ end of the LCa_v_β_B_ cDNA transcript was extended using 5′ RACE. Identified sequences were later confirmed by mRNA sequences from a published brain transcriptome of *Lymnaea stagnalis* [70], and unpublished genomic sequence spanning the LCa_v_β subunit made available by Daniel Jackson and Angus Davison (University of Nottingham). LCa_v_β subunit isoforms were cloned into mammalian expression vector pMT-2SX(R) using PCR inserts with flanking NotI and XhoI restriction sites spanning the start and stop codons of the LCa_v_β gene. DNA primers to create the 5′ end of the inserts using PCR were designed with a standard mammalian KOZAK sequence downstream of the Not1 site, **GCGGCCGC**CACC**ATG**G, where the underlined ATG sequence is the expected start codon. Final expression constructs in pMT-2SX(R) vector were confirmed for their expected DNA sequences by dideoxy terminator sequencing in both orientations (TCAG DNA sequencing facility, The Hospital for Sick Children, Toronto, Ontario).

### Sequence comparisons of calcium channel subunits

Multiple alignments and gene trees of sequences were generated in Phylogeny.fr [71]. The running average of similarity across Ca_v_β subunit sequences were generated with PLOTCON in EMBOSS [72]. Ca_v_β subunit sequences from differing exon 1a/1b, exon 2 and exon 7 splice isoforms in different organisms were identified by GenBank BLAST searches. The following are mostly full length Ca_v_β subunit sequences used for the generation of multiple alignments and gene trees shown in Figure 1 and S1 (*Genus species*, GenBank Accession #): Coanoflagellate (*Monosiga brevicollis*, XM_001748200.1), Poriferan (*Amphimedon queenslandica*, ACUQ01001402), Placozoan (*Trichoplax adhaerens*, ABGP01000238), Cnidarian (*Nematostella vectensis*, ABAV01020487), Lophotrochozoan (*Lymnaea stagnalis*, AF484087) and Ecdyzozyan (*Drosophila melanogaster*, U11074). The GenBank-derived Ca_v_1 channel sequences for Figure 1D were: Poriferan (*Amphimedon queenslandica*, XM_003382988), Placozoan (*Trichoplax adhaerens*, XM_002108894), Cnidarian (*Nematostella vectensis*, XM_001639004), Lophotrochozoan (*Lymnaea stagnalis*, AF484079) and human Ca_v_1.2 (BC146846). The GenBank-derived Ca_v_2 channel sequences for Figure 1D were: Placozoan (*Trichoplax adhaerens*, XM_002109739), Cnidarian (*Hydra magnipapillata*, XM_004210303), Lophotrochozoan (*Lymnaea stagnalis*, AF484082) and human Ca_v_2.1 (AB035727). Genomic and cDNA sequences for human Ca_v_β subunits were derived from NCBI Gene for CACNB1, CACNB2, CACNB3, CACNB4 genes. Genomic sequences for snail beta subunit were provided by Daniel Jackson and Angus Davison (University of Nottingham) and gaps in intron lengths were estimated from the genome sequence derived from the closely-related air-breathing, freshwater snail *Biomphalaria glabrata*.

### Calcium channel subunit expression in HEK-293T cells

Snail LCa_v_1 [15], [54] and LCa_v_2 channels [16], [17] from pond snail *Lymnaea stagnalis* were previously cloned and characterized in HEK-293T cells, expressed in pIRES2-EGFP bicystronic vector. Mammalian Ca_v_2.2 (α_1B_) channels were expressed in pMT2 expression vector, and co-transfected with pTRACER (EGFP). Positively-transfected channels were identified by green fluorescence with an Axovert 40 inverted CFL microscope using a GFP filter and mercury lamp excitation. Calcium channels were always co-transfected with mammalian α_2_δ_1_ subunit in pMT2 vector, with either no Ca_v_β subunits, mammalian Ca_v_β subunits (Ca_v_β_1b_ or Ca_v_β_2a_ or Ca_v_β3) or snail Ca_v_β subunit isoforms. Mammalian Ca_v_β and α_2_δ_1_ subunits were gifts from Gerald Zamponi (University of Calgary) and Terry Snutch (University of British Columbia).

### Quantitative RT-PCR of calcium channel subunits in snail tissues

mRNA expression was measured using quantitative Real-Time PCR (as previously published [14], [19], [73], [74]) using mRNA isolated from reproductively-active adult snails from *Lymnaea stagnalis* (shell lengths of 2.0 to 2.5 cm) illustrated in **Figure 4**. Adult versus sexually immature juvenile mRNA expression was compared in **Figure 4D**, where juveniles have shell lengths of 1.0 to 1.5 cm. qPCR primers sets (**Table S1**) were designed to selectively amplify LCa_v_β, LCa_v_1 and LCa_v_2 subunits using a universal primer set, and additional primer sets designed to amplify specific exons, such as N-terminal exons: LCa_v_β_A_ and LCa_v_β_B_ and differing exon 7 isoforms: LCa_v_β_−_ and LCa_v_β_+_. PCR primer specificity was confirmed by appropriate sized PCR products amplified from pooled template of cDNAs generated from freshly isolated mRNA and also cloned cDNAs. PCR primer efficiencies was then determined in relative standard curves using 1∶5 serial dilutions of pooled cDNA (1∶5, 1∶25, 1∶125; and 1∶625) as template for real time RT-PCR amplification. Triplicate reactions were carried out in 96-well PCR plates (Bio-Rad) for each dilution, with each well containing 0.5 µL of serially diluted cDNA, 5 µL of SsoFastTM EvaGreen Supermix (Bio-Rad), 0.5 µL of each 10 µM primer from a set, and 3 µL of water. PCR amplification, fluorescence reading, and melt curve analyses were carried out using a Bio-Rad C1000TM Thermal Cycler equipped with a CFX96TM Real-Time System and run by CFX Manager Software (Bio-Rad). All cycle threshold values used for analysis were determined relative to the average cycle threshold value of the control gene, HPRT1 (hypoxanthine phosphoribosyltransferase 1).

### Mammalian HEK-293T cell lines

We have described our optimized methods for the expression of ion channels in mammalian HEK-293T cells and their recording using whole-cell patch clamp in an online JoVE video journal [75]. Briefly, HEK-293T are cultured in Dubecco's Modified Eagle's Medium (DMEM, Sigma, #D5796) supplemented with 10% Fetal Bovine Serum (FBS; Sigma, #F1051) that had been heat-inactivated at 56°C for 30 minutes and 2.5 mL of Penicillin-Streptomycin solution (5000 units of penicillin and 5 mg streptomycin/mL; Sigma, #P4458). The complete HEK-293T media (500 mL) was further supplemented with 5 mL of 100 mM sodium pyruvate (Sigma, #S8636). In all instances complete culture media was heated to 37°C in a water bath before use.

### Subculturing of HEK-293T cells

To subculture cells, culturing media was removed from the flask and cells were washed twice with phosphate buffered saline (PBS; 6.70 mM KCl, 3.67 mM KH2PO4, 10.82 mM Na2HPO4-7H2O, 342 mM NaCl) that had been pre-warmed to 37°C. To detach cells, 1 mL of 0.25% Trypsin-EDTA (Sigma, #T4049) that had been heated to 37°C was added to the flask. The flask was then incubated at 37°C until the majority of cells had detached (to a maximum of five minutes). In the meantime, two new flasks were filled with 5.5 mL of fresh complete media (these will be used for the next passage). Also, flasks of cells for transfection were filled with 5 mL of fresh complete media. Once the cells were detached, 5 mL of fresh complete culture media was added and the cells were resuspended by pipetting up and down several times. The resuspended cells were then divided among the flasks so that each flask had a final volume of 6 mL, therefore the flasks to be used in the next passage were split 1∶12, while the flasks to be transfected were split 1∶6. These cells were then incubated under standard conditions. Cells were allowed to grow to 40–50% confluency prior to transfection.

### Transient transfection of HEK-293T cells using calcium phosphate precipitation

Transfection of HEK 293T cells was done using a calcium phosphate transfection protocol that was carried out by diluting 4 µg of each plasmid to be transfected in 30 µL of 2.5M CaCl_2_ and sterile milli-Q water to 300 µL. This was then added dropwise to 300 µL of 2× HES buffer (280 mM NaCl, 50 mM Hepes, 1.5 mM Na2HPO4-7H2O, pH 7.0). The mixture was then mixed well and allowed to incubate at room temperature for 20 minutes to allow for the formation of calcium phosphate crystals. During the incubation, media was removed from the flask to be transfected and replaced with 5.4 mL of fresh complete medium. After incubation, the calcium phosphate solution was added dropwise into the flask of cells. The flask was then incubated under standard conditions for eight to 16 hours. After eight to16 hours, the cells were washed twice with PBS pre-heated to 37°C and 6 mL of fresh complete medium was added. These cells were then incubated at 28°C in a humidified 5% CO2 atmosphere for two days before plating onto glass coverslips.

### Poly-L-Lysine coating of coverslips and plating cells onto coverslips

Prior to plating HEK cells, sterile, round glass coverslips (Fisher Scientific, #12-545-80) were coated in poly-L-lysine. The coverslips were spread out in a single layer at the bottom of a large (100 mm diameter) culture dish. A dilute poly-L-lysine solution was made by diluting 1.5 mL of 0.1% (w/v) poly-L-lysine (Sigma, #P8920) in 13.5 mL of milli-Q water. This solution was poured over the coverslips and they were allowed to incubate at room temperature for one hour. After one hour, the poly-L-lysine solution was removed and coverslips were washed twice in 15 mL of sterile water and then dried in a 56°C oven for two hours. After coating, coverslips were stored at 4°C for up to two weeks.

Two days after transfection, HEK cells were plated onto glass coverslips using the same method. Media was removed from the flask and the transfected cells were washed twice with PBS before the addition of 1 mL of trypsin, as in section 2.6.3. The cells were resuspended in 6 mL of complete media and added to 60 mm culture dishes containing the appropriate amount of complete media and four to six coverslips so as to give a split ratio of 1∶3 and 1∶4 for cells to be used in patch clamp experiments (HEK cells) and 1∶6 for cells to be used for antibody staining. These dishes were then incubated at standard conditions for three to four hours before they were moved to 28°C in a humidified 5% CO2 atmosphere.

### Whole cell patch clamp recording

Cells were recorded by whole-cell patch clamp method three to seven days after plating onto coverslips using an AxoPatch 200B amplifier, combined with a Digidata 1440A Data Acquisition System and pCLAMP 10 Software (Molecular Devices). Whole cell patch clamp recordings were carried out with an Patch pipettes for recording with pipette resistances of 2–5 MΩ, and with typical access resistance maintained after breakthrough between 4 and 6 MΩ. Only recordings with minimal leak (<10% of peak) and small current sizes (<500 pA) in HEK-293T cells were used due to loss of voltage clamp above 500 pA. Series resistance was compensated to 70% (prediction and correction; 10-µs time lag). For all recordings, leak subtraction was preformed offline and data was filtered using a 500 Hz Gaussian filter using Clampfit 10.2 software (Molecular Devices) before further analysis.

Calcium channel currents were measured in a 20 mM Ba^2+^ external bath solution: 20 mM BaCl_2_, 40 mM tetramethylammonium chloride, 10 mM glucose, 64 mM CsCl, pH 7.2) and patch pipettes were filled with internal solution (108 mM cesium methane sulfonate, 4 mM MgCl_2_, 9 mM HEPES, 9 mM ethylene glycol tetraacetic acid, pH 7.2). During all recording sessions, cells were maintained at a holding potential of −100 mV. Prior to recording, a test step to peak current (10 mV for LCa_v_1 or 30 mV for LCa_v_2) was performed to indicate if the cell was producing a current and if the state of the patch was suitable for gathering data. All data derived from patch clamp recording was statistically analyzed using one-way analysis of variation (ANOVA) tests online at http://www.danielsoper.com/statcalc3.

### Electrophysiology protocols for generating current-voltage relationships and steady-state inactivation

The current-voltage (IV) relationship was assessed by stepping cells from −100 mV to −50 mV for 450 ms and then increasing the voltage step by 10 mV in successive sweeps until reaching a potential of 50 mV. To analyze the effect of LCa_v_β subunits on the steady-state inactivation of the channels cells were stepped from −100 mV to peak voltage for 150 ms, then allowed to recover for 1 s before being held at −100 mV until all channels had reached inactivation (this conditioning step may take up to 15 seconds) then immediately stepping to peak again for 150 ms. In successive sweeps, the conditioning potential used to inactivate channels was increased by 10 mV each time until reaching 30 mV.

### Analyses of activation and current-voltage curves

To create activation and IV plots, current (I in pA) at each voltage step was normalized to peak current (I_max_ in pA) for that cell. Each normalized IV relationship was plotted and the individual reversal potentials (E_rev_; in mV) were determined by calculating the y-intercept of the linear portion of the IV curve (+20 to +40 for LCa_v_1; +30 to +50 for LCa_v_2). The reversal potential represents the membrane potential at which the driving force for Ca^2+^ (or Ba^2+^) influx is equal to the driving force for Ca^2+^ (or Ba^2+^) efflux and there is no net movement of Ca^2+^ (or Ba^2+^) through the calcium channels. The conductance (rate of ions flowing through the channel) was then determined for each voltage step using the equation: G = (I/I_max_)/(V−E_rev_), where G represents conductance (pS) of the channel and V represents the test voltage (mV). For each trace maximum conductance (G_max_) was then determined. The mean and standard error of the mean (s.e.m.) were calculated for (I/I_max_), E_rev_ and G_max_.

Activation plots were created using Origin 8 software (OriginLab, Northampton, MA) by importing IV data and then performing a Boltzmann transformation using the equation: Conductance (g) = I/(V−E_rev_). The Boltzmann-transformed data was then normalized and then a scatter of normalized activation versus voltage was created and curve fitted with the following Boltzmann equation: G/G_max_ = 1/(1+e((V−V_0.5_)/K_a_)), where V_0.5_ and K_a_ represent the half-activation voltage and slope factor of the activation curves, respectively. The half-activation potential is the voltage at which 50% of channels is expected to be open. The slope factor of the activation curve is a value that represents the delay of channels transitioning from the closed to active (open) position. The normalized activation data [(I/I_max_), V_0.5_, K_a_] were then averaged and the SEM was determined. The mean data was then plotted and a curve was simulated using the fitted parameters. IV plots were created by plotting voltage against mean (I/I_max_) ± SEM and then fitted with an Ohmic-Boltzmann curve using the following equation: I/I_max_ = G_max_(V−E_rev_)/(1+e^V−V0.5^/^Ka^).

### Analyses of steady-state inactivation

Steady-state inactivation values were determined by dividing the current after the inactivating (conditioning) pulse by the current prior to the inactivating pulse. Each data set was then imported into Origin and curve fit with the Boltzmann equation: (I/I_max_) = 1/(1+^e(V−V0.5)/Ki^) where V_0.5_ and K_i_ represent the half-inactivation voltage and the slope factor of inactivation, respectively. The half-inactivation potential is the voltage at which 50% of the channels are available and the other 50% of channels have transitioned from the open to the inactivated state. The values of V_0.5_ and K_i_ (determined using Origin 8) and (I/I_max_) were then averaged and the standard error of each means were calculated. The mean steady-state activation data was then plotted ± SEM and this data was used to simulate a Boltzmann curve derived from the fitted parameters. The relative inactivation of LCa_v_1 and LCa_v_2 currents were compared as the fraction of current remaining after 250 ms (R250).

### Western blotting of LCa_v_β subunits

LCa_v_β protein was identified on Western blots with rabbit anti- LCa_v_β subunit antibody made against KLH-coupled synthetic peptide with LCa_v_β sequence: SLDEEKEALRRET, which is downstream of the N-terminus and common to all LCa_v_β isoforms [20]. Western blots were prepared from protein homogenates isolated from HEK cell lysates, five days post-transfection. Total protein was separated by SDS-PAGE, and the contents of the gels were transferred onto 0.45 µM nitrocellulose membrane (Mandel, # W-10401196) overnight at 90 mA at 30 V. Before applying antibody, the nitrocellulose membrane must be blocked to reduce nonspecific interactions. Blocking buffer [10 mM Tris, 100 mM NaCl, 0.1% Tween-20(v/v), 5%(w/v) skim milk powder] was added to the membrane in a shallow dish (enough to completely cover the nitrocellulose), placed on a shaker and allowed to incubate at room temperature for two hours.

After blocking, the membrane was washed five times with Tween-Tris-Buffered Saline [TTBS; 10 mM Tris, 100 mM NaCl, 0.1% Tween-20(v/v)] for five minutes at room temperature. Next, the primary antibody (rabbit anti-LCa_v_β) was diluted 1∶2000 in blocking buffer and poured over the membrane after the last wash with TTBS. The primary antibody was incubated with the membrane overnight at 4°C. The following morning, the antibody was removed and the membrane was washed five times in TTBS for five minutes at room temperature, and then once in blocking buffer for 15 minutes at room temperature. After the second block, the secondary antibody (Goat anti-rabbit IgG coupled to horseradish peroxidase; Invitrogen, #65-6120) was diluted 1∶5000 in blocking buffer and added to the dish containing the membrane. The secondary antibody was incubated with the membrane for 30 minutes at room temperature before it was decanted. The membrane was then washed five times in TTBS for five minutes at room temperature. The membrane was then ready for chemiluminescent staining to detect the presence of LCa_v_β subunits.

LCa_v_β subunits were detected using the chemiluminescent stain ECL which was made by combining 20 mL of freshly prepared solution one (2.5 mM luminol, 396 µM p-coumaric acid, 100 mM Tris, pH 8.5) and 20 mL of freshly prepared solution two (100 mM Tris, 12 uL of 30% H_2_O_2_, pH 8.5). Solutions one and two were combined, poured over the membrane and incubated for one minute at room temperature. The blots were then exposed to x-ray film (Kodak, #819 4540) for one minute in a darkroom before development using an automated developer. Films were then compared to nitrocellulose membranes and the ladder was marked onto the film by hand.

### Potential caveats in studies of LCa_v_β subunits expressed in HEK-293T cells

We observe that snail LCa_v_β subunit isoforms promote membrane expression of calcium channels in HEK-293T cells, but the biophysical effects are relatively minor compared to the mammalian Ca_v_β subunits. A potential concern is a weak signal to background ratio perhaps due to a lack of saturation of heterologously-expressed Ca_v_β subunits in every cell recording that is also to contain calcium channels and a co-expressed α_2_δ subunit in a standard calcium-phosphate transfection protocol. Another issue is potentially contaminating, low levels of human calcium channels subunits reported in HEK-293T cells [76]. A fetal form of Cavβ3 subunit is detectable in Xenopus oocytes [77], [78], but little to no endogenous Cavβ subunits have been reported in HEK-293T cells [79]. We are confident that our data represents the snail Ca_v_β subunit isoforms effects on expressed snail calcium channels. Positively-expressing LCa_v_1 and LCa_v_2 HEK-293T cells corresponded largely to the green EGFP fluorescence of transfected cells, since EGFP and the calcium channels were derived from the same mRNA on bicistronic vector pIRES2-EGFP. LCa_v_β_A_ and LCa_v_β_B_ subunit expression can be identified by anti-rabbit LCa_v_β specific antibody in Western blots of transfected HEK cell lysates at appropriate protein sizes (**Figure S3**).

Expression of mammalian α_2_-δ subunits can cause dramatic shifts in voltage-sensitivities [7], [80], and perhaps the full contribution of molluscan Ca_v_β subunits will not be completely described without co-expression of native α_2_-δ subunits. Mining of molluscan genomes reveals three α_2_-δ subunits: a generalized one that resembles α_2_-δ_1_ and α_2_-δ_2_ subunit in structure, a second brain specific one that is a homolog to *Drosophila* straightjacket [81] and human α_2_-δ_3_ subunits, and a third more invertebrate specific α_2_-δ subunit.

## Supporting Information

Figure S1 **Multiple alignment of Cavβ subunits including those from a cnidarian (Nematostella), nematode (Caenorhabditis), ecdysozoan (Drosophila), lophotrochozoan (Lymnaea) and human gene isoforms (Cavβ1b, Cavβ2a, Cavβ3, Cavβ4b).** Highly conserved SH3 and GK domains are illustrated, also conserved secondary structures (α helices and β sheets), and calcium channel (AID) binding residues (blue residues) reported in crystal structures of Cavβ subunits. Exon boundaries (red lines) are indicated. Red boxes surrounding a base indicates that the intron splits between an amino acid base.
(TIF)

Figure S2 **Genomic sequences spanning exon 7 illustrating the alternate acceptor sites in** ***Lymnaea*** **snail calcium channel beta subunit, (LCavβ+ and LCavβ−) which generates a seven amino acid optional exon (7A+) or not exon (7A−).** Note that skipping of exon 7 generates a change in reading frame and truncated LCavβ. The frame shift occurs because exon 6 ends within a codon after the first nucleotide (phase 1), whereas the intron preceding exon 8 is located between codons (phase 0).
(TIF)

Figure S3 **Snail LCavβ - specific rabbit antibody localizes LCavβ_A_+ and LCavβ_B_+ proteins in transfected HEK cells by Western blotting.** Snail LCa_v_2 in pIRES2-EGFP vector were transfected alone (mock) or with coexpressed LCavβ_A_+ and LCavβ_B_+ plasmids in HEK cells. Expressed LCavβ_A_+ and LCavβ_B_+ proteins are identifiable on Western blots of transfected HEK cell lysates at appropriate size (62.8 kDa and 62.6 kDa), respectively.
(TIF)

Table S1 **Quantitative Real Time PCR (qPCR) primer sequence parameters used in** **Figure 4** **.**
(DOCX)
