# Supplementary figures and images for: Gene Splicing of an Invertebrate Beta Subunit (LCavβ) in the N-Terminal and HOOK Domains and Its Regulation of LCav1 and LCav2 Calcium Channels
Source: PLoS One. 2014 Apr 1;9(4):e92941. doi: 10.1371/journal.pone.0092941 (PMC3972191; doi:10.1371/journal.pone.0092941)

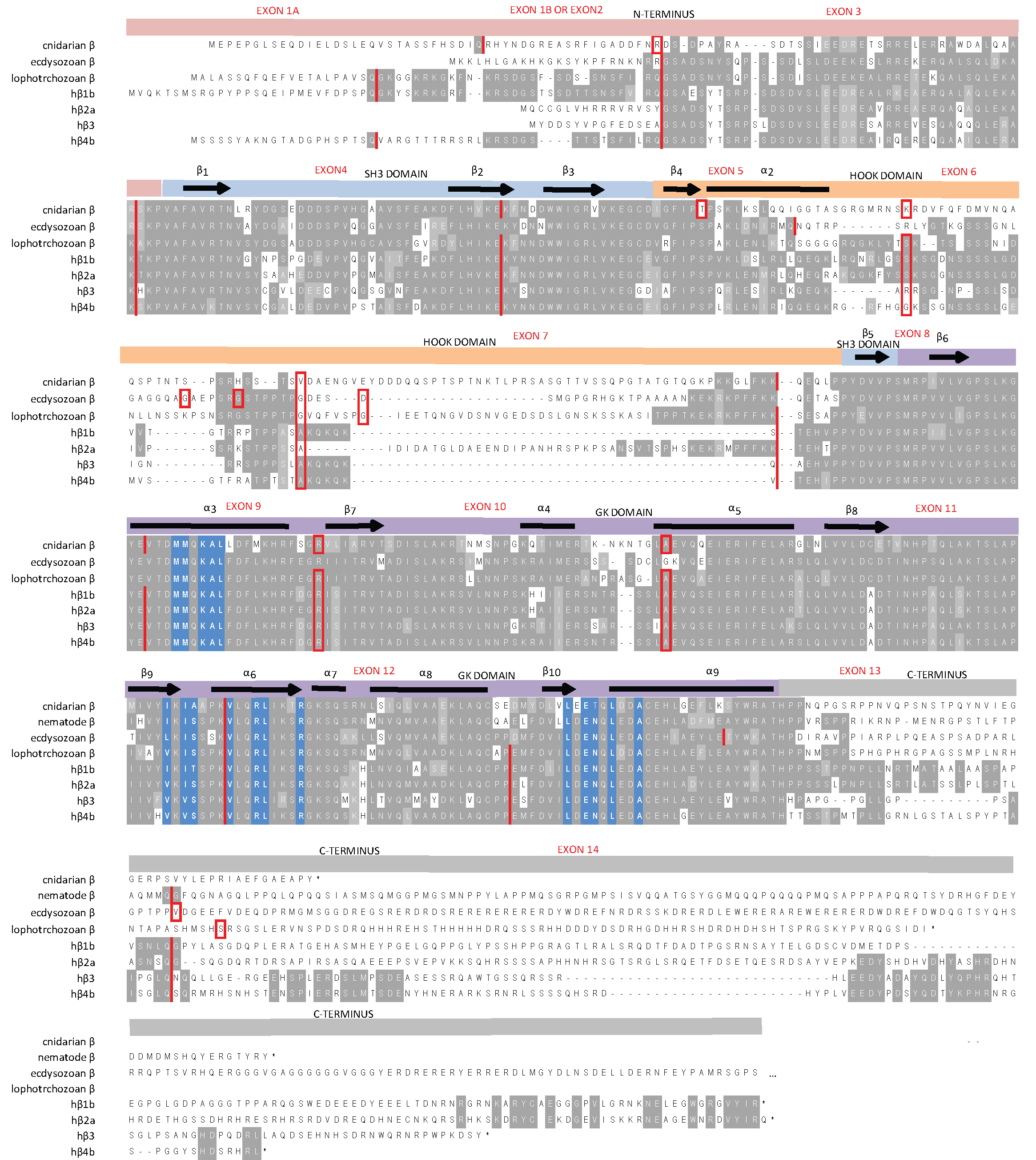

Supplement: Figure S1 — Multiple alignment of Cavβ subunits including those from a cnidarian (Nematostella), nematode (Caenorhabditis), ecdysozoan (Drosophila), lophotrochozoan (Lymnaea) and human gene isoforms (Cavβ1b, Cavβ2a, Cavβ3, Cavβ4b). Highly conserved SH3 and GK domains are illustrated, also conserved secondary structures (α helices and β sheets), and calcium channel (AID) binding residues (blue residues) reported in crystal structures of Cavβ subunits. Exon boundaries (red lines) are indicated. Red boxes surrounding a base indicates that the intron splits between an amino acid base. (TIF) [file pone.0092941.s001.tif]

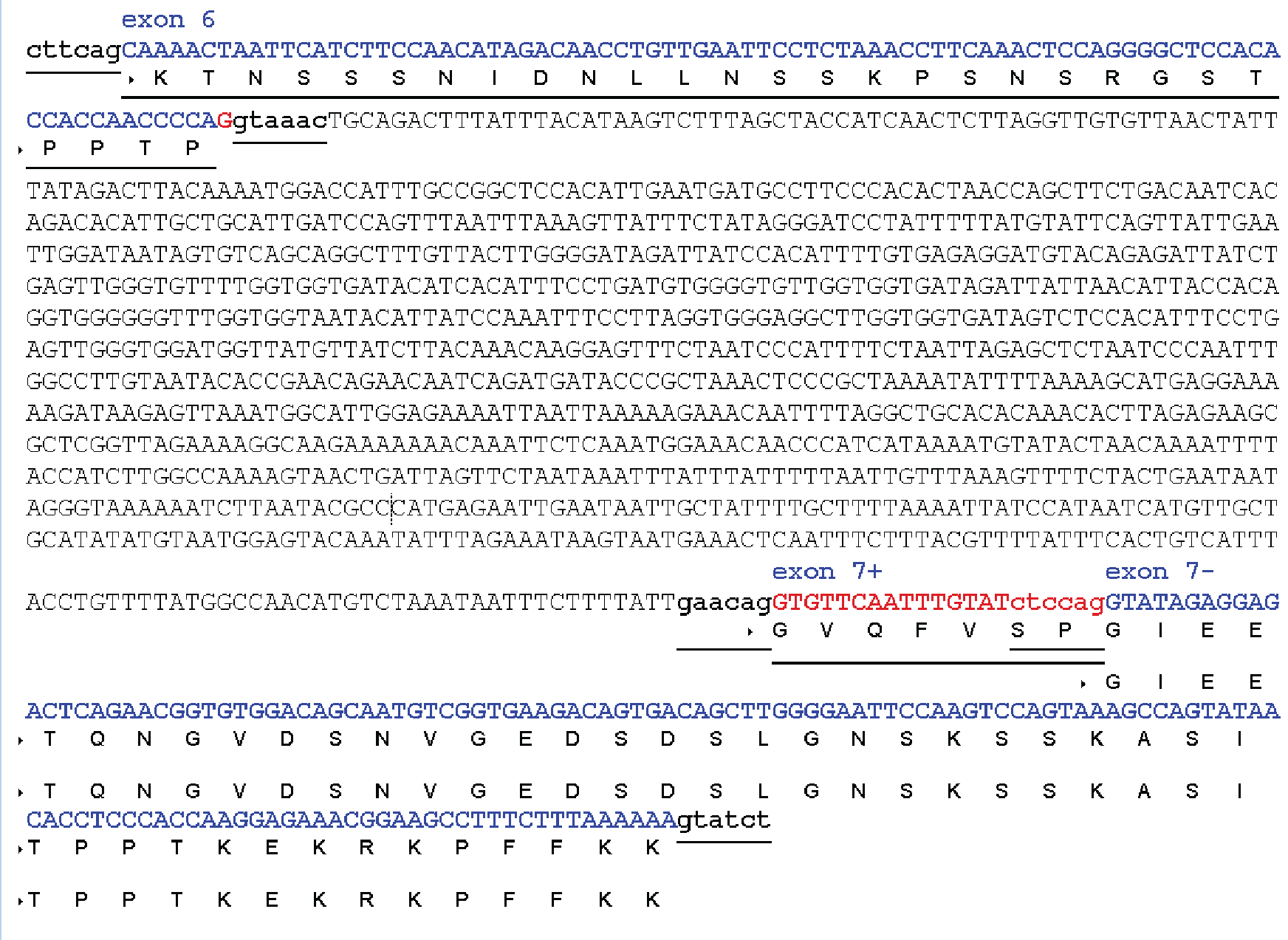

Supplement: Figure S2 — Genomic sequences spanning exon 7 illustrating the alternate acceptor sites in Lymnaea snail calcium channel beta subunit, (LCavβ+ and LCavβ−) which generates a seven amino acid optional exon (7A+) or not exon (7A−). Note that skipping of exon 7 generates a change in reading frame and truncated LCavβ. The frame shift occurs because exon 6 ends within a codon after the first nucleotide (phase 1), whereas the intron preceding exon 8 is located between codons (phase 0). (TIF) [file pone.0092941.s002.tif]

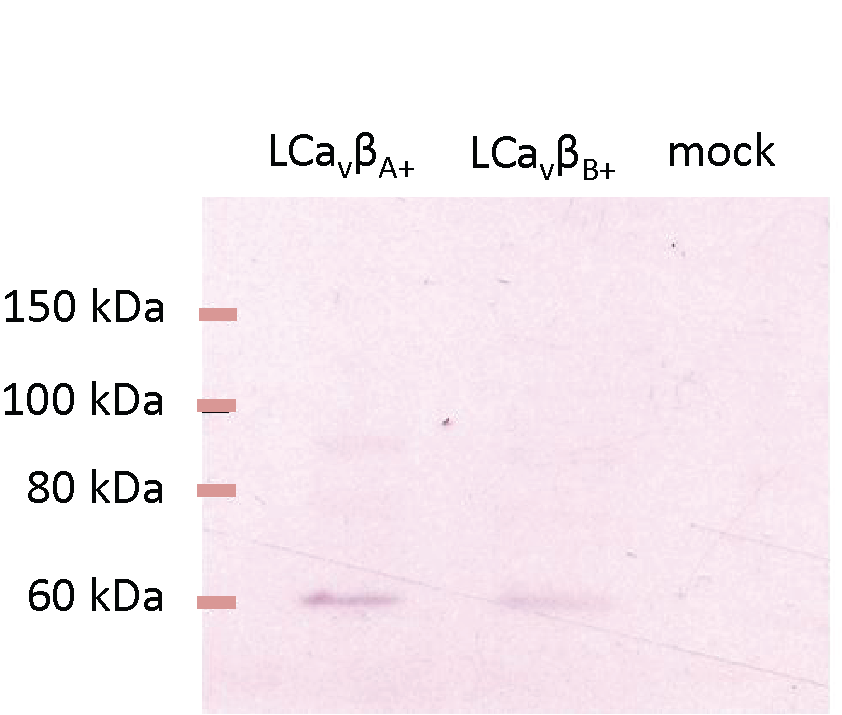

Supplement: Figure S3 — Snail LCavβ - specific rabbit antibody localizes LCavβA+ and LCavβB+ proteins in transfected HEK cells by Western blotting. Snail LCav2 in pIRES2-EGFP vector were transfected alone (mock) or with coexpressed LCavβA+ and LCavβB+ plasmids in HEK cells. Expressed LCavβA+ and LCavβB+ proteins are identifiable on Western blots of transfected HEK cell lysates at appropriate size (62.8 kDa and 62.6 kDa), respectively. (TIF) [file pone.0092941.s003.tif]
