# Supplementary material for: Gene Splicing of an Invertebrate Beta Subunit (LCavβ) in the N-Terminal and HOOK Domains and Its Regulation of LCav1 and LCav2 Calcium Channels
Source: PLoS One. 2014 Apr 1;9(4):e92941. doi: 10.1371/journal.pone.0092941 (PMC3972191; doi:10.1371/journal.pone.0092941)
Supplement: Table S1 — Quantitative Real Time PCR (qPCR) primer sequence parameters used in Figure 4 . (DOCX) [file pone.0092941.s004.docx]

| **Primer** | **DNA Sequence (5’ to 3’)** | **Amplification Length (bp)** | **Length (bp)** | **Tm (NN)** | **GC%** | **qPCR E** | **R2** | **Slope** |
| --- | --- | --- | --- | --- | --- | --- | --- | --- |
| Lymnaea HPRT1 5' | TGTAGAAGACATCATTGACACTGG | 145 | 24 | 53.86 | 42 | **90.4** | **0.985** | **-3.576** |
| Lymnaea HPRT1 3' | GCCAATATAATCTGGTGCGTAAC |  | 23 | 53.06 | 43 |  |  |  |
| LCav1 5' | CCTCATCATCATTGGCTCATT | 119 | 21 | 51.41 | 43 | **99.1** | **0.990** | **-3.347** |
| LCav1 3' | TCTCTCTCAGTTTCTGGAAGTCAC |  | 24 | 55.56 | 46 |  |  |  |
| LCav2 5' | TCTCGATGAATATGTTAGGGTCTG | 142 | 24 | 54.1 | 42 | **107.9** | **0.982** | **-3.147** |
| LCav2 3' | GTAGGCCAACTTGTAAGGACACTT |  | 24 | 54.86 | 46 |  |  |  |
| LCavβ Universal 5' | AGGAACATGAATGTCCAGCTAGT | 123 | 23 | 54.45 | 43 | **102.4** | **0.996** | **-3.265** |
| LCavβ Universal 3' | AAGAAACTCAGCTAAGTGCTCACA |  | 24 | 56.73 | 42 |  |  |  |
| LCavβ (+) 5' | GGCAAGGCAAGCTCTATACAA | 131 | 21 | 53.89 | 48 | **85.4** | **0.995** | **-3.731** |
| LCavβ (+) 3' | ACCTGGAGATACAAATTGAACACC |  | 24 | 52.92 | 42 |  |  |  |
| LCavβ (-) 5' | GGCAAGGCAAGCTCTATACAA | 119 | 21 | 53.89 | 48 | **91.7** | **0.993** | **-3.538** |
| LCavβ (-) 3' | CTCCTCTATACCTGGGGTTGG |  | 21 | 53.11 | 57 |  |  |  |
| LCavβ A 5' | CTTTGCCTGCTGTTTCTCAG | 126 | 20 | 53.35 | 50 | **106.0** | **0.990** | **-3.187** |
| LCavβ A 3' | TGTAGTTGGAATCCGCAGAA |  | 20 | 51.45 | 45 |  |  |  |
| LCavβ B 5' | GGAGAGCAATCCCATAATGC | 119 | 20 | 50.95 | 50 | **110.0** | **0.996** | **-3.104** |
| LCavβ B 3' | TGTAGTTGGAATCCGCAGAA |  | 20 | 51.45 | 45 |  |  |  |

Table S2 Quantitative Real Time PCR (qPCR) primer sequence parameters used in Figure 4
